# A systematic review on the effect of individual characteristics and management practices on equine cognition

**DOI:** 10.1007/s10071-025-02016-2

**Published:** 2025-11-26

**Authors:** Claire Ricci-Bonot, Kimberly Brosche, Paolo Baragli, Christine Nicol

**Affiliations:** 1 Animal Behaviour, Cognition and Welfare Group, School of Life Sciences, University of Lincoln, Lincolnshire, LN6 7TS UK; 2 Comparative Cognition Lab, Department of General Psychology, University of Padua, Via Venezia 8, Padova, 35131 Italy; 3 Department of Veterinary Sciences, University of Pisa, Viale delle Piagge 2, Pisa, 56124 Italy; 4 Research Center “E. Piaggio”, University of Pisa, Largo Lucio Lazzarino 1, Pisa, 56122 Italy; 5 The Royal Veterinary College, Hawkshead Lane North Mymms Hatfield, Hertfordshire, AL9 7TA UK

**Keywords:** Categorisation, Discrimination, Horse, Memory, Social learning, Spatial cognition

## Abstract

Equine cognition is relevant to the many roles that horses serve in society, such as leisure riding, competitions, or even animal-assisted therapy. Equine cognitive abilities have been explored in recent years. However, gaining an overview of horse cognition is challenging due to the broad range of abilities studied and the diverse methodologies employed. In addition, the subjects of existing equine cognition studies vary greatly in contextual factors such as their breed, age, sex, and management conditions – each of which may influence test performance in the following cognitive categories: Discrimination Learning; Learning Sets, Categorisation and Concept Formation; Spatial Cognition; Social Learning; and Memory. The aims of this review were (1) to establish whether contextual information was provided in research articles on horse cognition, (2) to tabulate information on the characteristics, housing, and management of subjects used in different categories of cognitive test, (3) to provide an overview of cognitive abilities demonstrated by horses, i.e., the results obtained in cognitive tests, with a specific emphasis on the contextual factors shaping them. The results of this review highlighted important points for future research. Better reporting of subject characteristics in scientific publications would enable investigation of the factors which shape horses’ cognitive abilities, and the use of standardized methods and procedures across studies would facilitate future comparative work.

## Introduction

Horses share a long history with humans (Levine 2005) and are of high societal relevance. The many roles of horses (Endenburg 1999) in society, such as leisure riding, competitions, or even animal-assisted therapy, demand strong learning abilities, high trainability and good memory from the animals. Still, much remains to be learned about the factors shaping their cognitive abilities which have been explored more widely only in recent years. For instance, horses can recognize adult humans and children cross-modally (Jardat et al. 2023b), successfully discriminate quantities in a match-to-sample task (Gabor and Gerken 2014), and learn to associate symbols with their blankets being put on or taken off (Mejdell et al. 2016). However, gaining an overview of horse (*Equus caballus*) cognition is challenging due to the broad range of abilities studied and the diverse methodologies employed. In addition, the subjects of existing equine cognition studies vary greatly in contextual factors such as their breed, age, sex, and management conditions each of which may influence test performance.

Studies of horses can also contribute to a comparative understanding of the effects of domestication on animal cognition. Horses are domesticated animals that occupy a niche between farm animals and companion animals (cf. Hausberger et al. 2019). In addition, horse breeds differ in selection purpose as much as individuals differ in their experience with humans. Therefore, studying horses alongside other domesticated animals can help establish whether the enhanced socio-cognitive abilities of companion animals (e.g. dogs and cats) in interaction with humans (e.g., Marshall-Pescini et al. 2017; Pongrácz et al. 2004, 2019; Téglás et al. 2012), are the result of (a) domestication alone, (b) specific selection for companionship, (c) individual life experience, or (d) a combination of these factors. In domesticated animals these abilities are known to surpass those of livestock (e.g., wild boars (*Sus scrofa scrofa*) and domestic pigs, Albiach-Serrano et al. 2012; dogs (*Canis familiaris*) and miniature pigs, Gerencsér et al. 2019; but see domestic pigs, Nawroth et al. 2014), thus comparing horses with each other – and with other domesticated animals – can provide valuable insights into the evolution of cognition more broadly.

Horses also exhibit remarkable social complexity which can shed light on potential links between sociality and advanced cognitive skills. The social brain hypothesis posits that life in social groups, and the challenges that come with it, have fuelled the evolution of larger brains and more sophisticated cognitive abilities (Dunbar 1998; Humphrey 1976; van Schaik and Burkart 2011; Whiten and Byrne 1988). Investigating horses could provide hints as to whether the relationship between social complexity and cognition found in primates (e.g., Krachun et al. 2019; Whiten and Byrne 1988) also holds in other phylogenetic lineages.

Despite the relevance and recent achievements of horse cognition research (Brubaker and Udell 2016; Hausberger et al. 2019), the specific contextual factors influencing equine cognition warrant further investigation. First, it remains unclear how methodological differences, which are prominent across equine studies, affect horses’ performance and, thereby, study outcome. Second, contextual factors, such as breed, age, sex, or management/welfare status might influence the results of equine cognition studies and are of fundamental interest for the reasons described next.

Breed differences may indicate how domestication and artificial selection have shaped horse cognition (cf. Price 1984). Domestication generally results in relaxed selection pressures on predator avoidance or foraging skills but increased selection for tameness, productivity, and/or successful interaction with humans, causing a shift in the allocation of energetic resources (Beilharz et al. 1993; Beilharz and Nitter 1998), including those allocated for cognition. For example, wolves (*Canis lupus*) and dogs show task-dependent differences in impulse control (Marshall-Pescini et al. 2015), diverging cooperation styles with humans (Range et al. 2019), and wolves outperform dogs when causal understanding is required (Lampe et al. 2017). In chickens, undomesticated red junglefowl (*Gallus gallus*) show enhanced individual learning, but a lower use of social information, relative to a domesticated layer breed (Lindqvist and Jensen 2009; Rutkauskaite and Jensen 2022). Equivalent studies in horses are not possible because the wild ancestor of horses is extinct (Bowling et al. 2003) and present-day feral horses are not inherently wild but rather the descendants of domestic horses (Orlando 2020). Domestic horses share a common ancestor with wild Przewalski horses but, while comparative studies of social behaviour have been conducted (Christensen et al. 2002) the cognition of these two sub-species has not yet been directly compared. Comparing more and less strongly selected breeds of domestic species is another approach to assess how artificial selection has affected cognition. For instance, chicken breeds differing in productivity diverge in performance in cognitive and memory tasks (Dudde et al. 2018; Rugani et al. 2023), cooperative dog breeds learn better from human demonstrations in a detour task than do independent breeds (Dobos and Pongrácz 2023) and heifers (*Bos taurus*) differ in performance in an observational learning task, potentially due to breed differences in fearfulness (Veissier 1993). In addition to genetic diversity, the way individual horses are worked varies greatly between breeds. That is, horse breeds selected and used for different purposes can be expected to vary in their cognitive abilities. Still, systematic investigations of breed effects on cognition in horses are scarce.

Age is another highly relevant determinant of cognitive abilities in humans and animals alike (Alexander et al. 2012; Erickson and Barnes 2003). During early development many cognitive abilities will be acquired. For instance, young dogs (56–973 days of age) become better at following human directional cues as they mature (Byosiere et al. 2023) and young pigs’ impulse control increases with age (Krause et al. 2021). However, the process of aging (time-related deterioration) can also influence cognition. Among non-human animals, the effect of aging on cognition has been particularly well studied in domestic dogs (for a review see: Chapagain et al. 2017; Szabó et al. 2016). These studies indicate that, with aging cognitive abilities such as memory and cognitive flexibility decline (Piotti et al. 2018, 2022; Tapp et al. 2003a, b; Watowich et al. 2020). Moreover, this negative effect of aging also seems to extend to learning and performance in a size discrimination task (Tapp et al. 2004), and in picture discrimination tasks (Wallis et al. 2016). Age effects on cognition, driven by development in young animals and/or aging in older individuals, can be expected also for horses but remain poorly understood.

Further, animals’ sex has long been an underestimated factor in research, including cognition research (Beery and Zucker 2011), despite being an important determinant of performance in cognitive tasks in farm animals (Bushby et al. 2018) and in horses and chickens (Koszałka et al. 2023). Numerous other studies have also reported differences in cognitive performance between sexes. Male sheep (*Ovis aries*) outperformed females in a reversal learning maze task (Erhard et al. 2004), while another study found females were faster (Hernandez et al. 2009). In a working memory task (Regolin et al. 2005): female domestic chicks performed better with social targets across different delays, while males excelled with non-social food rewards. In another bird species, the raven (*Corvus corax*), males were better at colour discrimination and object manipulation (Range et al. 2006). However, information on sex differences on equine cognition is sparse.

A closer investigation of the management conditions and welfare of the study subjects is also warranted because these factors can play a decisive role in cognitive development and in motivational and attentional aspects that affect performance. For instance, Bolhuis et al. (2004) found that enriched pigs are more distracted, despite also being more “optimistic” in cognitive bias tests (Asher et al. 2016). Especially in horses, a degree of social isolation and physical restriction is typical of many management systems, with negative welfare consequences (e.g. Chaya et al. 2006; Lesimple et al. 2019). Possibly, horses that are routinely restrained in riding or housing might give up avoiding negative stimuli altogether, including in future learning tasks (Hall et al. 2008), exhibiting so-called learned helplessness (dogs: Overmier and Seligman 1967; Seligman and Maier 1967). In addition, aversive and stressful experiences might cause horses to become apathic and exhibit depression-like symptoms (Fureix et al. 2012), which, in humans, are known to affect cognitive abilities such as memory (McDermott and Ebmeier 2009).

Despite increased efforts to probe equine cognition, the influences of breed, age, sex, and management/welfare on horses’ performance have remained largely understudied. A systematic review of the existing literature could remedy some of these shortcomings and identifying gaps by combining and critically analysing the results across studies.

The first aim of this review (Sect. 'Availability of contextual information') was to establish whether contextual information on subject characteristics, housing, and management was provided in research articles on horse cognition. The second aim (Sect. 'Tabulation of information on contextual information by field of cognition') was to tabulate information on the characteristics, housing, and management of subjects used in different categories of cognitive test. In line with previous reviews on horse cognition, we grouped these categories into: Discrimination Learning; Learning Sets, Categorisation and Concept Formation; Spatial Cognition; Social Learning; and Memory (Nicol 2002). The final aim was to provide a narrative overview of cognitive abilities demonstrated by horses, i.e., the results obtained in cognitive tests, with a specific emphasis on the contextual factors shaping them (Sect. 'Within-study effects of context' and 'Narrative review of results and consideration of between-study effects of context'). Determining whether these contextual influences affected performance in cognitive tests is very challenging but we approached this in two ways. First, we reviewed studies where the role of contextual factors had been included within the study design (Sect. 'Within-study effects of context'). Secondly, where test protocols were sufficiently similar, we compared results informally between studies with different contextual factors (Sect. 'Narrative review of results and consideration of between-study effects of context').

## Materials and methods

### Search strategies

The Web of Sciences database was used to search for research articles only (excluding reviews, editorials etc.) with the following inclusion criteria: in the title “horse* OR equid* OR pony OR ponies” and in the topic “cognition AND (abstract reason* OR category* OR discrim* OR social learn* OR concept format* OR spatial OR memor*)” on the 28/10/2024. The database provided 1954 articles. In the process of this research, there were no restrictions on the publication dates of the records.

### Selection of research studies: inclusion/exclusion criteria

**Fig. 1:**
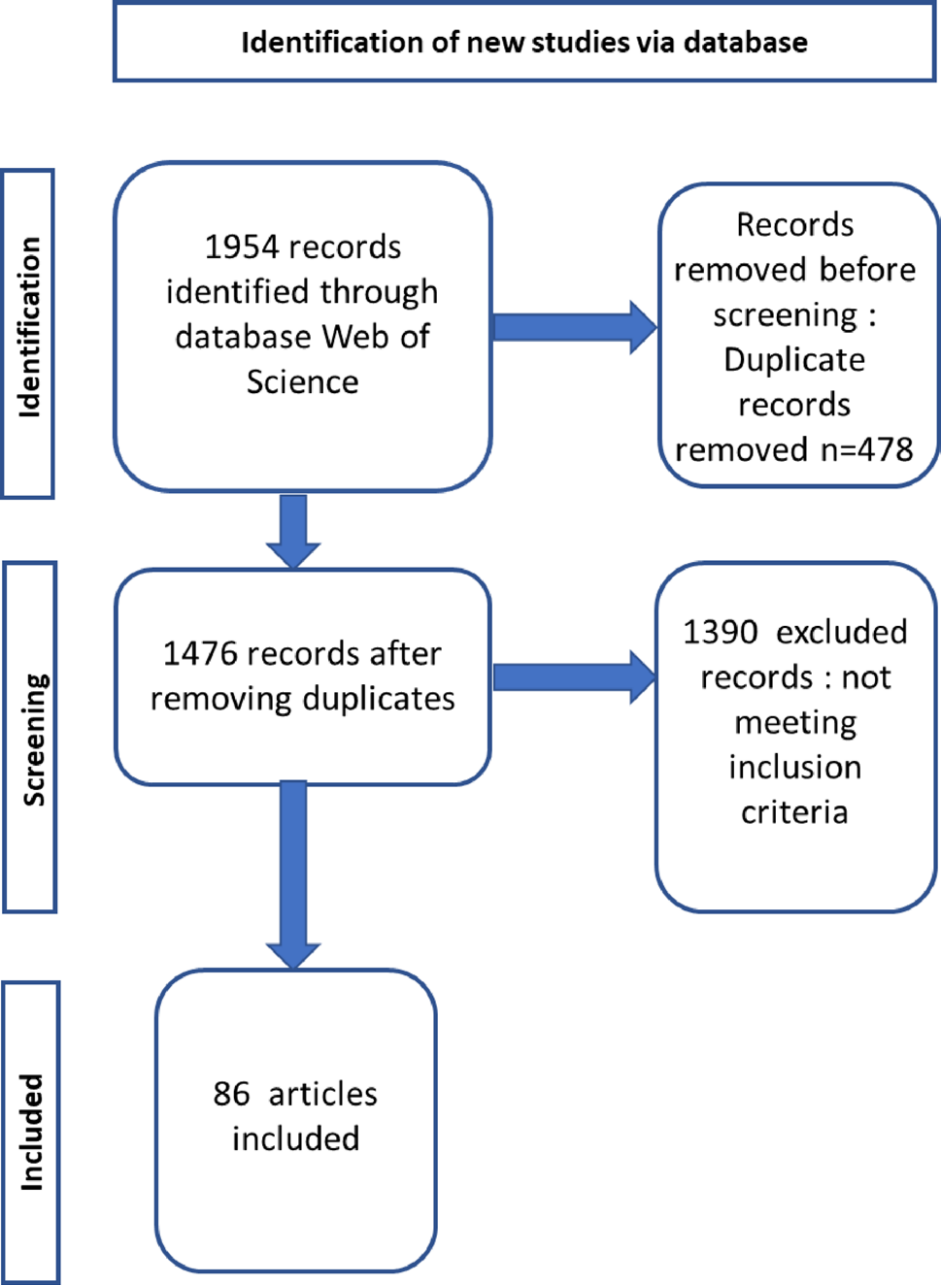
Flow diagram of selection process

During a first sift, all the duplicate records were removed. Then in a second sift, all the records which were not in English were removed. In a third sift, all the records which were not about horses (*Equus caballus*) were excluded. These were mainly recordings on animal species other than the horse (e.g. horse mackerel (*Trachurus trachurus*), horseshoe crab (*Limulus polyphemus*), greater horseshoe bat (*Rhinolophus ferrumequinum*), lesser horseshoe bat (*Rhinolophus hipposideros*), Chinese horseshoe bat (*Rhinolophus sinicus*)), or plant species (e.g. horse chestnut (*Aesculus hippocastanum*), Japanese horse chestnut (*Aesculus turbinata*), horse weed (*Conyza canadensis*)), and articles which used expressions such as ‘Putting the cart before the horse’ and ‘Trojan Horse’. Then, all the records which were not articles, such as books, book chapters, reviews, commentaries, meeting records, conference abstracts were excluded from this review as they were still present despite the selection on Web of Science to restrict the search to articles only. Finally, all the studies included were scientific articles on complex cognition in horses, i.e. on discrimination, abstract reasoning, categorisation, concept formation, spatial cognition, social learning and memory. The process and number of items excluded at each stage is shown in Fig. 1.

### Information from selected articles

Information related to contextual information: subject characteristics (breed, sex and age), housing and management, were extracted from the selected articles. For management, the information collected concerned practices related to housing, feeding and amount of work under saddle (i.e. the frequency or duration of time that a horse is ridden). For housing, the important information was whether the horse lived in a stable or in an indoor barn with or without access to a paddock, in a pasture (size of housing and time spent over 24 h) and whether the horse lived alone or with conspecifics. When subject horses lived alone we searched whether they could have contact with others (visual or touch). We collected information on whether the horses were fed *ad libitum* (e.g. grazing at pasture, hay) or at fixed timing (e.g. pellets). We also looked at the time spent by the horses working under the saddle.

## Results

### Availability of contextual information

**Fig. 2:**
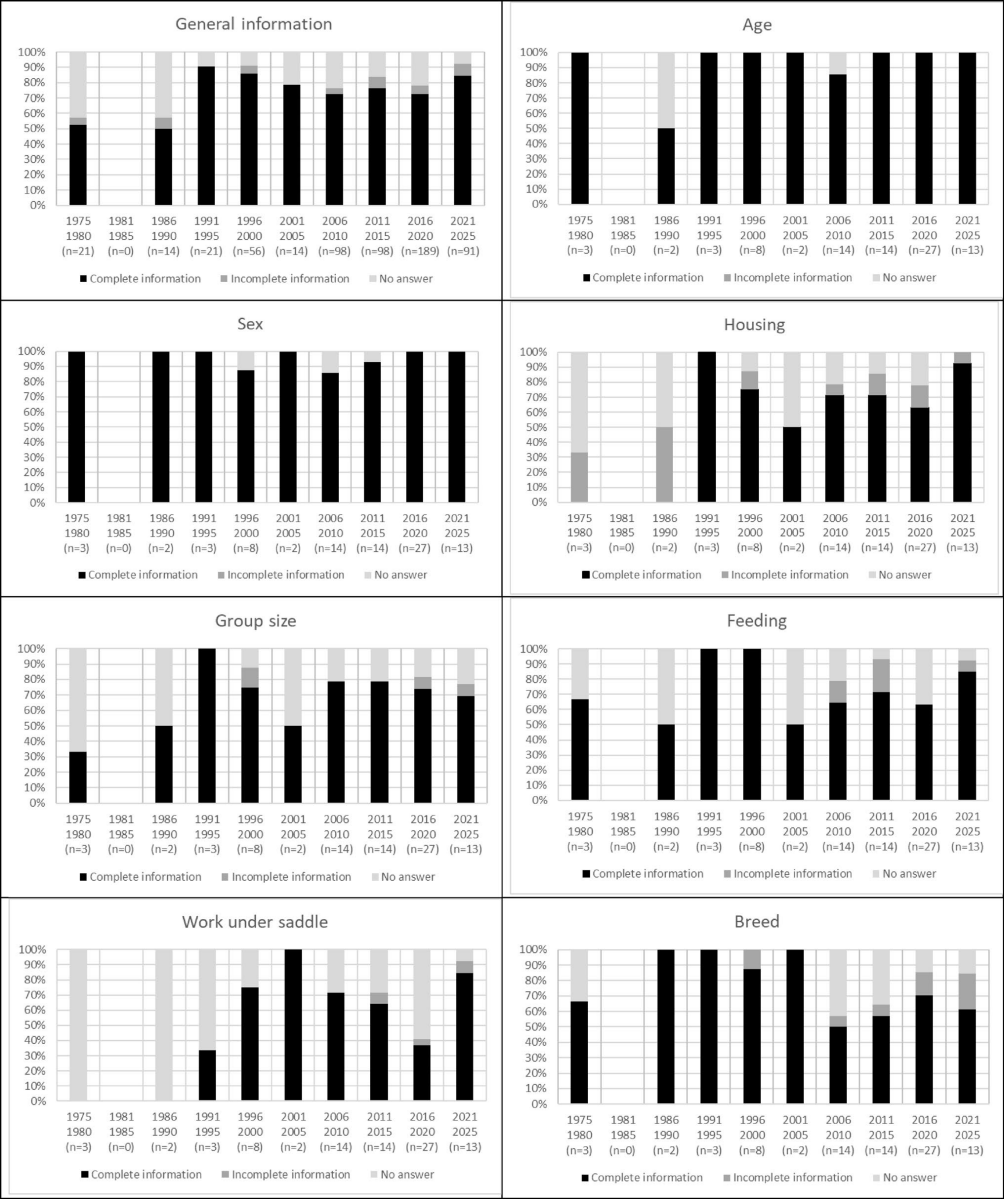
Evolution of the information present in the 86 studies for the following six categories: age, sex, housing, group size, feeding, work under saddle and breed. ‘General information’ corresponds to the six categories pooled together

For this systematic review, 86 articles were selected and analysed. The oldest articles dated from 1979 and the most recent from 2024. From 1991 onwards, the data provided in the studies are increasingly complete (Fig. 2). While age and sex have always been relatively well reported, the results of this analysis confirm a clear improvement in the information reported from 1991 onwards for housing and group size. A similar trend for improved information on feeding and working under saddle of the horses was not confirmed and even deteriorated from 2001 for feeding, and from 2006 for working under saddle. The breeds were always well documented between 1986 and 2005. However, since 2006 this information tends to be absent.

**Fig. 3:**
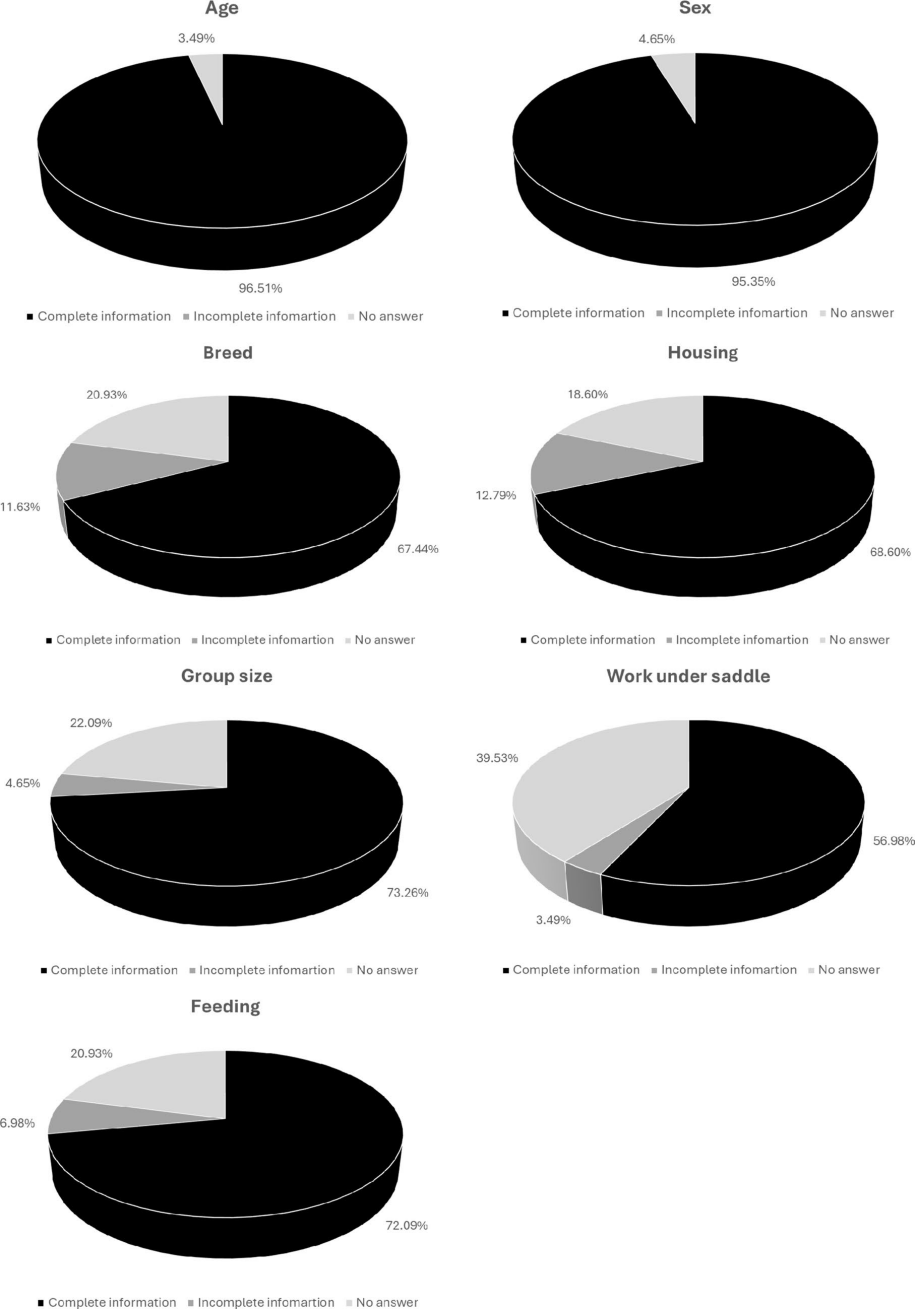
Information obtained for the 77 articles about age, sex, breed, housing, group size, work under saddle and feeding

Out of the 86 articles, subject age was mentioned 96.51% (*n* = 83) and sex 95.35% (*n* = 82) of the time while breed was indicated in 67.44% of cases (*n* = 58). In 25 studies the horses used in the experiments were of at least 2 different breeds (Fig. 3).

The housing of the horses was specified in 68.60% of cases (*n* = 57) and was mostly pasture with other conspecifics (37 groups). For horses living alone in stables (23 groups of subjects), more than half of these groups had access to the paddock/pasture (14 groups out of 23) with at least one conspecific (5 out of 14). Only one study mentioned that when the horses are alone in their stable, they can have visual and nose contact with the others. Two other studies mentioned that horses had a period of socialisation. The various studies provide very little information on the size of the housing and the time spent in it. For work under saddle, the researchers indicated this information in 56.98% of cases (*n* = 49), with 36 articles where the horses were working under saddle against 13 where it was not the case. The time spent working was very rarely indicated. Finally, food was specified in 72.09% of cases (*n* = 62), with 58 fed *ad libitum* and/or 35 fed at fixed times.

### Tabulation of information on contextual information by field of cognition

This section will focus on information present across the different studies on contextual information by field of cognition, i.e. discrimination learning, learning sets, categorisation and concept formation, spatial, social learning and memory, presented in Tables 1, 2, 3, 4 and 5 respectively.

#### Discrimination learning (Table 1)

Table 1 shows that discrimination learning has been examined by many authors, with two differing primary aims. First, research has been conducted to investigate whether particular stimuli can be discriminated, generally using food as a conventional reward. These studies include those using simple visual stimuli such as coloured markings on feed buckets, coloured markings on a panel, cross versus circle on a black or white background, symbols of different shapes and sizes and quantities. Second, more recent studies have used a far broader range of stimuli including equine vocalisations or human emotions.

For the 28 studies included in Table 1, unlike the other cognitive categories, contextual information was often missing, including for breed (9 studies), housing (9 studies), group size (10 studies) and ridden status (13 studies). Eleven studies examined only one breed, of these Welsh ponies were used in four studies. The age of the horses tested varied considerably: from 10 months to 32 years (not tested in the same study). Most studies were conducted on horses of more than one sex/reproductive status, that lived alone in stables but various sexes, with some time spent out in a paddock. In five studies, horses lived in groups in a pasture.

**Table 1:** Information from the different studies on discrimination learning

Discrimination between	Locomotion				Sociality	Feeding		Subject characteristics			Articles
	Time (over 24 h) spent in				Group size	Ad libitum	Fix timing	Breed	Sex	Age	
	Stable	Indoor barn	Pasture/Paddock	Work under saddle							
Quantities	Yes	No	n.a.	Yes (some of them)	*n* = 1	n.a.	n.a.	n.a.	*F*,* M*	*4–30 years*	Balestrieri et al. (2024)
	No	No	Yes	n.a.	*n* ≥ 2	Yes	Yes			*3–23 years*	
Colours	Yes	No	Yes	Yes	n.a.	Yes	Yes	Various breeds	F, G	8–22 years	Evans et al. (2024a)
Human emotions	No	Yes	Yes (4 h)	No	*n* ≥ 2	Yes	No	Welsh ponies	F	6 years	Jardat et al. (2024)
Objects	*Yes*	*No*	*Yes (night and/or part of day)*	Yes (3–7 h per week)	*n* = 1 (stable), *n* ≥ 2 (pasture)	Yes	No	12 breeds	F, M	*x̄ age*: *10.6 years*	Kappel et al. (2023)
							Yes			*x̄ age*: *16.6 years*	
Letters	No	No	Yes	Yes (for 2 horses)	*n* = 5	Yes	Yes	Garranos	F, G	2–13 years	Schubert et al. (2022)
Colours, sizes, shapes and patterns	No	No	Yes	No	*n* ≥ 2	Yes	No	Danish Warmblood	*F*,* M*	10–12 months	Christensen et al. (2021)
Intra- and inter-specific faces	Yes	No	Yes (6–8 h)	n.a.	*n* = 1	No	Yes	Franches-Montagnes horses	F, M	x̄ age: 11.2 years	Ragonese et al. (2021)
Human faces	No	Yes	Yes	n.a.	*n* ≥ 2	Yes	No	Welsh ponies	F	3 years	Lansade et al. (2020b)
Human emotions	Yes	No	n.a.	Yes	*n* = 1	n.a.	n.a.	Arabian and thoroughbreds	F, S, G	x̄ age: 3.6 years	Sabiniewicz et al. (2020)
Horse emotions	No	Yes	Yes	n.a.	*n* ≥ 2	Yes	No	Welsh ponies	F	x̄ age: 8.47 years	Trösch et al. (2020c)
Colours and shapes	n.a.	n.a.	n.a.	n.a.	n.a.	n.a.	n.a.	5 breeds	*F*,* S*,* G*	*10–25 years*	Briefer Freymond et al. (2019)
Human vocalisations	n.a.	n.a.	n.a.	Yes	n.a.	n.a.	n.a.	n.a.	F, G	7–22 years	Smith et al. (2018)
Symbols	Yes (night)	No	Yes (day)	Yes	*n* = 1 (night), *n* = 2–3 (day *)	Yes	Yes	*10 breeds*	F, G	3–16 years	Mejdell et al. (2016)
Horse emotions	n.a.	n.a.	n.a.	n.a.	n.a.	n.a.	n.a.	n.a.	*F*,* G*	*3–32 years*	Wathan et al. (2016)
										*5–27 years*	
Sizes and shapes	n.a.	n.a.	n.a.	Yes (for 2 horses)	n.a.	Yes	n.a.	n.a.	F, M	1–9 years	Tomonaga et al. (2015)
Quantities	No	No	Yes	n.a.	*n* = 3	Yes	No	Shetland	F, G	6–13 years	Gabor and Gerken (2014)
Horse body odours	Yes (4 × 4 m)	No	n.a.	Yes (3 h)	*n* = 1	Yes	Yes	n.a.	*F*,* G*	7–17 years	Péron et al. (2014)
Colours and shapes	No	No	Yes	n.a.	*n* = 2, *n* = 6, *n* = 8	Yes	n.a.	3 breeds	F	8–19 years	Gabor and Gerken (2010)
Horse body odours	Yes (for 8 horses)	No	No	No	*n* = 1	Yes	Yes	Various breeds	F	*5–17 years*	Hothersall et al. (2010)
	No	Yes (for 2 horses)	No		*n* = 2						
Human faces	n.a.	n.a.	n.a.	n.a.	n.a.	Yes	Yes	n.a.	n.a.	n.a.	Stone (2010)
Horse sounds	n.a.	n.a.	n.a.	n.a.	n.a.	n.a.	n.a.	Colombian fine-step horses	n.a.	5 years	Cruz-Becerra et al. (2009)
Quantities	Yes	No	Yes	n.a.	*n* = 1	Yes	Yes	n.a.	F, M	n.a.	Uller and Lewis (2009)
Colours and positions	n.a.	n.a.	n.a.	Yes	n.a.	Yes	Yes	Thoroughbreds	F, G	15–18 years	Miyashita et al. (2000)
Colours	No	No	Yes	n.a.	*n* = 17	Yes	Yes (except during exp. period)	*3 breeds*	*F*,* G*	*1–2 years*	Sappington et al. (1997)
Colours	n.a.	n.a.	n.a.	n.a.	n.a.	Yes	Yes	Thoroughbreds and Standardbred	*F*,* G*	4–6 years	McCall (1989)
Horse body odours	Yes	No	n.a.	n.a.	*n* = 1	n.a.	n.a.	American Saddlebred	*F*,* S*,* G*	n.a.	Marinier et al. (1988)
Colours and shapes	Yes (some of them)	Yes (some of them)	n.a.	n.a.	*n* = 1 or *n* = 2	No	Yes	*Quarter horse*	F, S	*1–17 years*	Mader and Price (1980)
								*Thoroughbred*	F	*1–22 years*	
Positions and brightness	n.a.	n.a.	n.a.	n.a.	n.a.	Yes	Yes	Quarter horse and Quarter horse x Thoroughbred	*F*,* S*,* G*	1 year	Fiske and Potter (1979)

(F = female, G = gelding, M = male, S = stallion, x̄ = mean, * = except one horse, italic = taken into account in the statistical analyses)

#### Learning sets, categorisation and concept formation (Table 2)

Table 2 summarises background information for the 27 studies that considered the cognitive ability of horses to acquire information, form concepts, and find solutions in the absence of prior training (Nicol 2002), as well as studies on concept generalization.

More than half of the horses used in these studies lived in groups on pasture (16 studies). In seven studies the horses lived in a stable alone, three providing some time in a paddock. Some studies provided no information on housing (2 studies), group size (4 studies), breed (7 studies), sex (1 study), ridden status (6 studies and 1 incomplete information), or feeding (3 studies). Information was provided on age (which varied from 10 months to 38 years) and breed (with most studies conducted on warmbloods and ponies). In this review, we used the term “hotblood horses” for horses which have sensitive, nervous, and excitable temperament (generally, it corresponds to Arabian or Thoroughbred horses). Then “coldblood horses” for horses which on the opposite are generally placid (e.g. draft horses). “Warmblood horses” (also commonly described as sport horses) have been bred from both hot and cold-blooded lines over multiple generations to perform with various equestrian sports.

**Table 2:** Information from the different studies on learning sets, categorisation and concept formation

Test	Locomotion				Sociality	Feeding		Subject characteristics			Articles
	Time (over 24 h) spent in				Group size	Ad libitum	Fix timing	Breed	Sex	Age	
	Stable	Indoor barn	Pasture/Paddock	Work under saddle							
Inhibitory task	Yes	No	Yes	Yes	n.a.	Yes	Yes	Various breeds	F, G	11–22 years	Evans et al. (2024b)
Concept formation	No	Yes	Yes (12 h)	No	*n* ≥ 2	Yes	No	Welsh ponies	F	x̄ age: 7.9 years	Jardat et al. (2023a)
Concept formation	No	Yes	Yes	No	*n* ≥ 2	Yes	No	Welsh ponies	F	x̄ age: 8.9 years	Jardat et al. (2023b)
Concept formation	Yes	No	Yes	n.a.	*n* = 1	Yes	Yes	Various breeds	F, S, G	x̄ age: 11.3 years	Trösch et al. (2020a)
Concept formation	*Yes* *(3.4 × 3.3 m)*	*No*	*No*	Yes (3–4 h)	*n* = 1	Yes	No	Various breeds	F, G	8–17 years	d’Ingeo et al. (2019)
	*No*	*No*	*Yes* *(1-2ha)*	n.a.	*n* = 2–4				F, S, G	2–22 years	
Abstract reasoning	Yes	No	Yes (1–3 h alone + 1–3 h in group)	Yes	*n* = 1 or *n* ≥ 2	Yes	Yes	Warmbloods and ponies	*F*,* G*	*7–25 years*	Esch et al. (2019)
Categorisation	No	Yes	Yes	No	*n* ≥ 2	Yes	No	Welsh ponies	F	x̄ age: 9.47 years	Trösch et al. (2019a)
Concept formation	Yes	No	No	Yes	*n* = 1	Yes	Yes	Saddle horses	F, S, G	x̄ age: 10 years	Trösch et al. (2019b)
Abstract reasoning	Yes * (night)	n.a.	Yes * (day)	n.a.	*n* ≥ 2 *	n.a.	n.a.	*3 breeds*	*F*,* M*	x̄ age: 15.44 years	Haemmerli et al. (2018)
Categorisation	n.a.	n.a.	n.a.	n.a.	n.a.	n.a.	n.a.	n.a.	F, G	x̄ age: 14 years	Nakamura et al. (2018)
Concept formation	No	No	Yes (12 ha)	n.a.	*n* = 10	Yes	No	4 breeds	F, G	10–24 years	Baragli et al. (2017a)
Concept formation	Yes	No	No	Yes	*n* = 1	No	Yes	Thoroughbreds	F, G	6–19 years	Ringhofer and Yamamoto (2017)
Concept formation	Yes	No	Yes (3 times a week)	n.a.	*n* = 1	Yes (pasture)	Yes	Various breeds	F, G	x̄ age: 14.9 years	Lovrovich et al. (2015)
Concept formation	No	No	Yes	Yes (except during exp. period)	*n* = 7	Yes	No	Shetland	F, G	3–12 years	Gabor and Gerken (2012)
Concept formation	No	No	Yes	Yes	*n* ≥ 2	Yes	Yes	n.a.	*F*,* G*	*8–15 years*	Lampe and Andre (2012)
Concept formation	n.a.	n.a.	Yes	Yes	n.a.	Yes *	n.a.	n.a.	*F*,* G*	*1.6–31 years*	Proops and McComb (2012)
					n.a.				*F*,* S*,* G*	*2–25 years*	
Categorisation	No	No	Yes	Yes	*n* = 11	Yes	No	3 breeds	F, G	8–16 years	Hanggi (2010a)
Concept formation	n.a.	n.a.	n.a.	Yes	n.a.	n.a.	n.a.	n.a.	G	5–28 years	Kundey et al. (2010)
Concept formation	*No*	*No*	*Yes*	Yes *	*n* ≥ 2	Yes	Yes *	n.a.	*F*,* G*	*10 months – 38 years*	Proops and McComb (2010)
Concept formation	*n.a.*	*n.a.*	Yes *	Yes *	*n* ≥ 2 *	Yes *	n.a.	n.a.	F, G	*3.5–38 years*	Proops et al. (2010)
Concept formation	No	No	Yes	Yes *	app. 30	Yes	Yes*	n.a.	*F*,* G*	*3–29 years*	Proops et al. (2009)
Categorisation	No	No	Yes (3 ha)	No	*n* = 24	Yes	No	Danish Warmblood	S	2 years	Christensen et al. (2008)
Concept formation	No	No	Yes	Yes	*n* = 8	Yes	Yes	3 breeds	G	4–7 years	Hanggi (2003)
Categorisation	No	No	Yes	Yes	*n* = 5	Yes	Yes	Paint horses and Anglo-arabian	G	2 ½−16 years	Hanggi (1999)
Concept formation	n.a.	n.a.	Yes	Yes	*n* = 3	Yes	Yes	Quarter horses and Thoroughbreds	n.a.	13–16 years	Flannery (1997)
Categorisation	No	No	Yes	Yes	*n* = 4	Yes	Yes	Purebred Arabian	F, G	11–22 years	Sappington and Goldman (1994)
Categorisation	Yes (3.7 × 2.7 m)	No	No	n.a.	*n* = 1	Yes	Yes	Quarter horses	F, G	8–26 years	Dougherty and Lewis (1991)

(F = female, G = gelding, M = male, S = stallion, x̄ = mean, * = some of them, italic = taken into account in the statistical analyses)

#### Spatial cognition (Table 3)

Table 3 summarises background information for the 8 studies that considered spatial cognition. Most of them focused on detour tasks (5 out of 8 studies; Table 3). Further detail is given in Sect. 'Narrative review of results and consideration of between-study effects of context'.

Moreover, these works provided little contextual information (see Table 3). Indeed, in 3 out of 8 studies, the researchers did not mention the housing conditions of the horses. However, contextual information was provided by Briefer Freymond et al. (2020) who used horses from 19 different farms but due to the larger scope of the study, horses were placed into broad contextual categories e.g. for housing, horses were categorised as living alone or in groups, in stable or in pasture.

No information was given on work under saddle, except for the research of Janczarek et al. (2023). Mal et al. (1993) did not mention it, however the horses were less than 5 months old. Regarding the sociability of the horses, three studies did not provide any information on the size of the group. In the other studies, horses mostly lived in groups of more than two individuals.

Information on the characteristics of the subjects was provided most of the time, with all studies indicating the age (from 4.5 months to 31 years, not tested in the same study), seven studies indicating the sex (mostly females) of the horses studied and six their breed (with mainly warmblood horses).

**Table 3:** Information from the different studies on Spatial cognition

Task	Locomotion				Sociality	Feeding		Subject characteristics			Articles
	Time (over 24 h) spent in				Group size	Ad libitum	Fix timing	Breed	Sex	Age	
	Stable	Indoor barn	Pasture/Paddock	Work under saddle							
Maze	Yes	No	Yes (4 h)	Yes (2 h daily, 5 days a week)	n.a.	Yes	Yes	Hucul horses	F	x̄ age: 9.2 years	Janczarek et al. (2023)
Detour	n.a.*	n.a.*	n.a.*	n.a.	*n* = 1 or *n* ≥ 2	n.a.	n.a.	12 breeds	*F*,* S*,* G*	*3–24 years*	Briefer Freymond et al. (2020)
Solve visible and invisible displacement	No	Yes	Yes	n.a.	*n* ≥ 2	Yes	No	Welsh ponies	F	x̄ age: 6.60 years	Trösch et al. (2020b)
										x̄ age: 7.90 years	
Detour	No	No	Yes (40 × 20 m)	n.a.	*n* ≥ 2	Yes	No	Standardbred horses	F	*x̄ age*: *10.8 years*	Baragli et al. (2017b)
Detour	n.a.	n.a.	n.a.	n.a.	n.a.	n.a.	n.a.	n.a.	n.a.	10–31 years	Osthaus et al. (2013)
Detour	No	No	Yes (75 × 75 m)	n.a.	*n* = 10	Yes	No	Italian saddle horses	F	x̄ age: 9.5 years	Baragli et al. (2011)
Grid	Yes (3 × 3 m)	No	No	n.a.	*n* = 2	Yes	Yes	Arabian	F, S	4.5 months	Mal et al. (1993)
Detour	n.a.	n.a.	n.a.	n.a.	n.a.	n.a.	n.a.	n.a.	F, S, G	2–16 years	Haag et al. (1980)

(F = female, G = gelding, S = stallion, x̄ = mean, * = horses from 19 different locations with different housing, italic = taken into account in the statistical analyses)

#### Social learning (Table 4)

Early studies of equine social learning primarily focused on intra-species learning, i.e. the use of a trained conspecific horse as demonstrator, whereas more recent studies have started to investigate inter-species learning between horse and human (Table 4).

All the research conducted on social learning provided information about the sex and age of the horses, and more than half provided information on the breeds of the horses studied (8 out of 11 studies) with three of them focusing only on Icelandic horses (Table 4). In most cases information on feeding was provided (7 out of 11 studies). Six studies mentioned whether horses were working under saddle and one study provided partial information, i.e. the information was not specified for all groups of horses tested. Information on horse housing was mostly provided (8 out of 11 studies) with approximately half of the horses living in stables alone and going out to a paddock alone or with conspecifics and the other half living all the time in paddock. The horses used in these studies mostly had contact with other horses (6 out of 11 studies). It should be noted that one study did not provide any information on housing, sociability, feeding and breed.

**Table 4:** Information from the studies on social learning

Learn from	Locomotion				Sociality	Feeding		Subject characteristics			Articles
	Time (over 24 h) spent in				Group size	Ad libitum	Fix timing	Breed	Sex	Age	
	Stable	Indoor barn	Pasture/Paddock	Work under saddle							
Humans	*n.a.* *	*n.a.* *	*Yes* (3–4 h)	n.a.	*n* ≥ 2	n.a.	n.a.	36 breeds	*F*,* G*	*2–14 years*	Bernauer et al. (2020)
Humans	Yes (3 × 4 m)	No	Yes (8 h min.)	Yes	*n* = 1 (stable)	Yes	Yes	Icelandic horses	*F*,* S*,* G*	*4–18 years*	Rorvang et al. (2020)
Humans	n.a.	n.a.	n.a.	Yes	n.a.	n.a.	n.a.	n.a.	F, G	*7–25 years*	Henriksson et al. (2019)
Humans	Yes (3.5 × 3.5 m)	No	Yes (several time per week)	Yes	*n* = 1	Yes	Yes	Various breeds	F, G	4–19 years	Burla et al. (2018)
Horses	No	No	Yes	n.a.	Gr1: *n* = 14, Gr2: *n* = 12, Gr3: *n* = 20 + 1 mule	Yes	No	4 breeds	F, G	x̄ age: Gr1 = 3 years, Gr2 = 12 years, Gr3 = 17 years	McVey et al. (2018)
Humans	n.a.	n.a.	Yes (3–4 h)	n.a.	*n* ≥ 2	n.a.	n.a.	Various breeds	F, S, G	*3–12 years*	Schuetz et al. (2017)
Horses	No	No	Yes (24 h, 5 ha)	No	*n* = 12	Yes	No	Icelandic horses	F, G	3 years	Rorvang et al. (2015a)
Horses	No	No	Yes (24 h, exp. 1 and 2: 5 ha, exp. 3: 0.6–1.6 ha)	No	Exp. 1 and 2: *n* = 12, Exp. 3: *n* = 4	Yes	No	Icelandic horses	Exp. 1 and 2: F and G, Exp. 3: F	Exp. 1 and 2: 3 years, Exp. 3: 2 and 9 years	Rorvang et al. (2015b)
Horses	No	No	Yes (24 h, 8 ha)	n.a.	*n* = 23	Yes	No	Danish Warmblood	G	2–3 years	Ahrendt et al. (2012)
	No	Yes	Yes	Yes	*n* ≥ 2	n.a.	n.a.	At least 4 breeds	*F*,* G*	*3–18 years*	
	Yes	No	Yes	Yes	*n* = 2–10	n.a.	n.a.				
Horses	Yes	No	Yes	Yes	*n* = 1	Yes	No	*12 breeds*	*F*,* G*	*3–28 years*	Lindberg et al. (1999)
Horses	Yes	No	Yes	n.a.	*n* = 1 (stable)	Yes	No	5 breeds	F, G	2–20 years	Clarke et al. (1996)

(F = female, G = gelding, S = stallion, exp = experiment, Gr = group, min = minimum, x̄ = mean, * = horses from 22 different locations with different housing, italic = taken into account in the statistical analyses)

#### Memory (Table 5)

Table 5 summarises background information on the memory capacity of horses which has been investigated across various tasks such as delayed-response, discrimination, instrumental and spatial learning (Table 5).

Most of the studies conducted on the memorization abilities of horses were conducted on horses living in groups (8 out of 12 studies) (Table 5). As for the social learning, all the studies provided information on the sex and age of the horses tested. Only one study did not provide information on the housing of the horses. Regarding the breeds of horses tested, three studies did not provide any information on this aspect (two studies wrote various breed and the last one did not mention anything). It is noted that three studies were conducted on Welsh ponies only.

**Table 5:** Information from the different studies on memory

Task	Locomotion				Sociality	Feeding		Subject characteristics			Articles
	Time (over 24 h) spent in				Group size	Ad libitum	Fix timing	Breed	Sex	Age	
	Stable	Indoor barn	Pasture/Paddock	Work under saddle							
Spatial	Yes (3.6 × 3.6 m)	No	Yes (8 h only weekend)	Yes	*n* = 1 (stable)	Yes	No	Various breeds	F, G	x̄ age: 14.9 years	Greening et al. (2021)
Spatial	No	No	Yes	Yes	*n* ≥ 2	n.a.	n.a.	n.a.	F, G	5–14 years	Whishaw and Burke (2021)
Discrimination	No	Yes	Yes	n.a.	*n* ≥ 2	Yes	No	Welsh ponies	F	3 years	Lansade et al. (2020a)
Discrimination	No	No	Yes	n.a.	*n* = 7	Yes	No	Shetland	F, G	7–14 years	Gabor and Gerken (2018)
Delayed-response	No	Yes (half a day, 6 × 4 m)	Yes	No	*n* ≥ 3	Yes	No	Welsh ponies	F	x̄ age: 7.2 years	Valenchon et al. (2013a)
Instrumental	No	Yes (6 × 4 m)	Yes (4 h)	No	*n* ≥ 3	Yes	Yes	Welsh ponies	F	x̄ age: 6 years	Valenchon et al. (2013b)
Delayed-response	No	No	Yes	Yes	*n* > 4	Yes	No	4 breeds	G	10–14 years	Hanggi (2010b)
Instrumental	No	Yes (winter)	Yes	No	*n* = 23, *n* = 5–6 (winter)	n.a.	n.a.	Anglo-Arabian and French Saddlebred	F, G	1 year	Sankey et al. (2010)
Discrimination, Categorisation	No	No	Yes	Yes	*n* = 12	Yes	No	3 breeds	G	13 years	Hanggi and Igersoll (2009)
Delayed-response	n.a.	n.a.	n.a.	Yes	n.a.	n.a.	n.a.	4 breeds	F, G	3–22 years	McLean (2004)
Instrumental	Yes	No	Yes (1 day per week)	Yes (5–15 h per week)	*n* = 1 (stable), *n* > 2 (pasture)	Yes	Yes	*Various breeds*	*F*,* G*	3–17 years	Le Scolan et al. (1997)
Instrumental	Yes (half a day)	No	Yes	No	*n* = 1 (stable), *n* > 2 (pasture)	No	Yes	French Saddlebred	*F*,* M*	*1–3 years*	Wolff and Hausberger (1996)

(F = female, G = gelding, M = male, exp = experiment, x̄ = mean, italic = taken into account in the statistical analyses)

### Within-study effects of context

In this Section we look at within-study information on contextual factors. Where authors have mentioned the role of contextual factors within their own studies we highlight this. We then look deeper within the literature to identify whether such information might exist for future analyses. We looked for individually-identifiable data on horse performance in cognitive tests alongside horse characteristics.

#### Discrimination learning

Authors examining discrimination learning have occasionally investigated contextual factors such as breeds, age and sex. Among the studies that used more than one breed, only 3 out of 11 investigated the effect of breed on discrimination test results (Mader and Price 1980; Mejdell et al. 2016; Sappington et al. 1997). Mader and Price (1980) found that Quarters learned faster than the Thoroughbreds and Mejdell et al. (2016) that the warmblood horses needed fewer days of training (11.1 days) than cold-blood horses (11.6 days).

Sappington et al. (1997) found the opposite result, with no effect of breed on performance in a discrimination task, but they noted a tendency for a breed difference in the first reversal criterion for the mean number of successes. However, it is important to note that the discrimination task was not the same between these three studies. Specifically, the horses had to discriminate between coloured markings on feed buckets (Sappington et al. 1997), coloured markings on a panel (Mader and Price 1980) or symbols (Mejdell et al. 2016).

In 8 out of the 20 studies that used subjects of different age researchers did not find an effect of age on the discrimination performance (Balestrieri et al. 2024; Briefer Freymond et al. 2019; Hothersall et al. 2010; Sappington et al. 1997; Wathan et al. 2016). However, Sappington et al. (1997) noted that a greater proportion of 2-year old horses than 1-year old horses (yearlings) completed 100% of the reversals. Kappel et al. (2023) found older horses (mean age ± SD: 14.8 ± 5.7) had a better learning ability than younger ones (mean age ± SD: 10.6 ± 2.51). In an older study, age negatively correlated with performance (Mader and Price 1980).

In 9 out of the 21 studies that used subjects of different sexes, 7 found no effect of sex on results (Balestrieri et al. 2024; Briefer Freymond et al. 2019; Christensen et al. 2021; McCall 1989; Péron et al. 2014; Sappington et al. 1997; Wathan et al. 2016). Wathan et al. (2016) noticed, however, that males spent more time looking at visual stimuli (pictures of horse face) than females during discrimination testing, whereas females spent more time avoiding the visual stimuli and were more reactive. Two studies found a significant effect of subjects’ sex on their results (Fiske and Potter 1979; Marinier et al. 1988). Fiske and Potter (1979) found a correlation between trainability scores and learning ability indices for males but not for females.

Whether or not authors had directly considered contextual factors, these factors were measured in some studies and we extracted additional information on this. For example, we found information on breed and age from seven studies (Cruz-Becerra et al. 2009; Gabor and Gerken 2010; Kappel et al. 2023; Lansade et al. 2020b; Mader and Price 1980; Mejdell et al. 2016; Schubert et al. 2022) and only age for three others (Miyashita et al. 2000; Tomonaga et al. 2015; Wathan et al. 2016), as these authors provided lists of individual horse characteristics alongside individually-identifiable horse performance data. However, Mader and Price (1980) did not link all results with the subjects’ characteristics. Among the six other studies where the age and breed of the subjects were provided as well as identifiable horse performance data, two used horses of the same age and breed (Cruz-Becerra et al. 2009; Lansade et al. 2020b). For the four remaining studies, two used only four or five horses of the same breed with different ages making it difficult to analyse the possible effect of age on the results (4 equids: Gabor and Gerken 2010; 5 equids: Schubert et al. 2022), one study had 17 different breeds for 27 horses making it difficult to perform statistical analysis (Kappel et al. 2023) and the other one, by Mejdell et al. (2016) had already investigated the effect of breed as described above. However, with the information provided by the authors it would also be possible for future researchers to test the effect of age on the results. Regarding the studies that mentioned the age of each individual alongside their results, one tested the effect of age (Wathan et al. 2016; see above), while the other two used only three horses in their experiments (Miyashita et al. 2000; Tomonaga et al. 2015). For sex, it was possible to extract information from seven studies (Gabor and Gerken 2010, 2014; Kappel et al. 2023; Mejdell et al. 2016; Miyashita et al. 2000; Schubert et al. 2022; Tomonaga et al. 2015). But except for Mejdell et al. (2016) and Kappel et al. (2023), the five other studies used five or fewer horses in their experiment (3 horses: Gabor and Gerken 2014; Miyashita et al. 2000; Tomonaga et al. 2015, 4 horses: Gabor and Gerken 2010 and 5 horses: Schubert et al. 2022) making it difficult to investigate an effect of sex on their results. Moreover, it is also difficult to see a possible effect of sex on the research of Mejdell et al. (2016) as the authors indicate the identities of subjects linked with the training process of the 10 different steps across 14 days but used 18 geldings and only 5 females. Kappel et al. (2023) provided individually-identifiable horse performance data which could be used in future analyses.

Finally, in term of housing, Kappel et al. (2023) found that horses from one of the two riding schools used for their research needed more trials to meet the criteria. Surprisingly, the horses from this riding school were older (14.8 ± 5.7) than the other ones (10.6 ± 2.51) and had a higher welfare score based on a welfare assessment protocol (looking at housing, access to conspecific and animal-based measurements for example).

#### Learning sets, categorisation and concept formation

Among the 27 studies that looked at learning sets, categorisation or concept formation, authors occasionally investigated contextual factors. For example, only one investigated the contextual factor breed and found no effect on their results (Haemmerli et al. 2018). Age and sex were examined in 6 studies each (age and sex: Esch et al. 2019; Lampe and Andre 2012; Proops and McComb 2010, 2012; Proops et al. 2009; age: Proops et al. 2010; sex: Haemmerli et al. 2018). Two studies found that younger horses were more reactive than older ones, without specifying exact ages, during presentation of stimuli, with longer duration of first look during incongruent trials (Lampe and Andre 2012) and a faster reaction to the playback (Proops and McComb 2012: 0–5 years). The other studies found no effect of age on their results (Esch et al. 2019; Proops and McComb 2010; Proops et al. 2009, 2010). Proops and McComb (2010, 2012) also found an effect of sex on the results of their two studies, with females performing better at choosing the attentive experimenter than geldings (Proops and McComb 2010) and also they were better than males at recognising specific handlers (Proops and McComb 2012). The other contextual factor which has been examined in three studies is the housing or more precisely the difference of location between horses used within the same research (d’Ingeo et al. 2019; Proops and McComb 2010; Proops et al. 2010). Only d’Ingeo et al. (2019) found an effect of the housing on their results, with horses living alone in a stable being more sensitive to the valence of the acoustic stimuli (such as behaviours related to frustration during the negative valence stimulus) than horses living with conspecifics in a pasture. This effect was not found by Proops and McComb (2010) but all their horses were living in a pasture with conspecifics. The difference between the two populations in this study was the degree of interaction with humans.

We attempted to identify if data was potentially available for future analyses, even if authors had not examined these effects themselves. It was possible to extract information retrospectively about subjects’ characteristics such as breed, sex and/or age alongside individually-identifiable horse performance data in 9 studies (Baragli et al. 2017a; Dougherty and Lewis 1991; Flannery 1997; Gabor and Gerken 2012; Hanggi 1999, 2003, 2010a; Nakamura et al. 2018; Sappington and Goldman 1994). However most of these research used 4 or fewer subjects in their research (2 subjects: Hanggi 1999, 2003; 3 subjects: Flannery 1997; 4 subjects: Baragli et al. 2017a; Dougherty and Lewis 1991; Hanggi 2010a; Sappington and Goldman 1994;), except one study which used 7 horses (Gabor and Gerken 2012), making it difficult to perform statistical analysis on these datasets. Only Nakamura et al. (2018) used a larger number of subjects (*n* = 19). The authors indicated the sex of each subject but they tested 18 geldings and only one female, making it impossible to investigate an effect of sex on their results.

#### Spatial cognition

Of the 8 studies that looked at spatial cognition, 3 studies provided very little information on the housing and size of the group, and only one provided information about the work under saddle as previously showed in Table 3. Moreover, only two studies tested the impact of one of the contextual factors examined in this review: age (Baragli et al. 2017b; Briefer Freymond et al. 2020). Researchers from these studies did not find any effect of the subjects’ age on their results.

Briefer Freymond et al. (2020) also examined the impact of sex and did not find any effect of this contextual factor on their results. For the seven remaining studies, four only used females (Baragli et al. 2011, 2017b; Janczarek et al. 2023; Trösch et al. 2020b) and one did not indicate subjects’ sex (Osthaus et al. 2013).

Henriksson et al. (2019) noted a difference in detour task performance according to the subjects’ size. Indeed, in their experiment they used 11 horses and 11 ponies. Approximatively half of the ponies were successful in performing the detour with or without the human demonstration (45.45%) whereas the horses were mostly successful after the human demonstration (36.36%). Only one horse succeeded in performing the task with or without the human demonstration.

Despite the fact that all studies indicated the age of horses, it was impossible for us to extract this information retrospectively in most articles, as only Haag et al. (1980) provided a list of individual horse characteristics such as age (2–16 years) and sex (females, stallions and geldings) alongside individually-identifiable horse performance data. This information revealed that age certainly had no impact on the results as the best performing horse in a maze test, according to the number of days before three consecutive right choices (if several horses had the same number of days, researchers used the mean time through maze), was a 3-year-old female followed by a 16-year-old gelding. The least successful horse was a 2.5-year-old male. However, to confirm this observation, age and sex characteristics should be tested separately as in the study of Haag et al. (1980), the two males were aged 2 and 2.5 years and were classed respectively in the maze at rank 8 and 10 out of 10.

#### Social learning

Social learning between horses has not been easy to identify conclusively in the research presented below. For social learning between horse and human the results are mixed with some positive results on the ability of human to act as demonstrator in the research of Schuetz et al. (2017) and Bernauer et al. (2020).

Authors examining social learning have occasionally investigated contextual factors. Among these 11 studies, 6 investigated the influence of horse age on social learning (Ahrendt et al. 2012; Bernauer et al. 2020; Henriksson et al. 2019; Lindberg et al. 1999; Rorvang et al. 2020; Schuetz et al. 2017). While most of the research did not find an effect of age on their results, Lindberg et al. (1999) showed that young horses spent more time performing investigative behaviour than older horses, whereas Henriksson et al. (2019) found that older horses looked more at the food bucket than younger ones, but neither of these studies specified precise ages. In a study by Krüger et al. (2014), which was not captured in this review and drawn to our attention at a later stage, researchers found that younger horses were able to learn to open a feeding device from watching older horses demonstrate the behaviour. However, the authors only mentioned that learners were between 2 and 15 years old, without specifying ages. The effect of breed has only been investigated by Lindberg et al. (1999), who found that non-warmbloods were the most successful in learning, reaching the criterion more rapidly than warmbloods and opening an operant device more often. Four studies examined the effect of sex on social learning performance but found no effect of this contextual factor (Ahrendt et al. 2012; Bernauer et al. 2020; Lindberg et al. 1999; Rorvang et al. 2020).

We were able to extract the subjects’ sex in two studies as the researchers provided a list of individual horse with this information alongside individually-identifiable horse performance data (Henriksson et al. 2019; Schuetz et al. 2017). In the study of Henriksson et al. (2019), half of the equines which successfully performed the detour task with or without the human demonstration were female (3/6) but only one female succeeded after the human demonstration out of the 5 equines. Schuetz et al. (2017) found that of the 8 horses out of 12 that reached the learning criteria, 5 were females, 2 were geldings and 1 stallion. The 4 horses which did not reach the learning criteria were 3 geldings and 1 female. Bernauer et al. (2020) were the only ones to investigate the potential effect of type of management on their research and found that this had no impact on the horses’ performance.

Only 3 studies out of 11 provided a list of individual horse characteristics alongside individually-identifiable horse performance data (Bernauer et al. 2020; Henriksson et al. 2019; Schuetz et al. 2017). While we know that the horses used in Henriksson et al. (2019) were between 7 and 25 years old, the age of the horses is not indicated individually.

#### Memory

Among the 12 studies, only two examined contextual factors and no effect of sex or age (Wolff and Hausberger 1996) or breed and sex (Le Scolan et al. 1997) was found on memory test performance. However, Wolff and Hausberger (1996) noticed that younger females (1-year-old) learnt an instrumental task more quickly and they were also more successful at learning a spatial task than males.

It was impossible for us to extract information about subject characteristics, breed and age, retrospectively as most of the studies (9 out of 12) did not provide a list of individual horse characteristics alongside individually-identifiable horse performance data. The 3 studies which provide this information (Gabor and Gerken 2018; Hanggi 2010b; Hanggi and Ingersoll 2009) had only tested a maximum of four horses, therefore making it difficult to attribute inter-individual differences to individual characteristics. Indeed, in the research of Hanggi (2010b), the four horses were geldings of different breeds (Arabian, Draft mix, Grade horse and Missouri Foxtrotter), 10-year old, except one 14-year-old horse. In some cases, only one horse has been tested, as was the case in Hanggi and Ingersoll (2009) who used one horse for the categorization memory test (a 13-year-old Paint gelding) and another one for the size concept memory test (a 13-year-old Arabian gelding).

### Narrative review of results and consideration of between-study effects of context

Because in the previous Sect. 'Within-study effects of context', it was not generally possible to determine the role of contextual factors on cognitive capabilities of horses this section takes a different approach. A narrative review of the results of research studies on the different categories of cognitive test is followed by an informal consideration of the influence of contextual factors where confounding factors were not too extreme. It was not possible to compare results between studies using quantitative or statistical methodologies, as too few studies looked at a single cognitive paradigm, too few studies reported the relevant information, and most studies used different methodologies even to investigate the same problem.

#### Discrimination learning

Within the overall category of Discrimination learning, a first group of studies has examined whether horses can distinguish physical cues (e.g. size, colour, shape, quantities, positions), sometimes using these procedures as the basis for further investigation of the general reversal learning abilities of horses. In their research, Fiske and Potter (1979) used a Y-maze with spatial and colour cues to test reversal learning discrimination in horses (Fiske and Potter 1979). The yearling subjects were Quarter horses and Quarter horses crossed with Thoroughbreds who were required to discriminate between two buckets to obtain a food reward. Over the 21 days of experiments, the mean number of errors as well as the mean number of trials to reach criterion decreased emphasising a learning process was used by the horses. However, in the experiment of Sappington et al. (1997), most of the horses could not achieve the reversal learning more than one time. There are a large number of factors that might explain this difference including the design of the experiment (the position of the buckets was alternated in Fiske and Potter (1979) whereas the position of the bucket was randomly changed in Sappington et al. (1997), the various breeds used by Sappington et al. (1997) or housing (Fiske and Potter 1979 did not mention any information about the housing of their horses. Sappington et al. (1997) mentioned that the 17 subjects lived together in a pasture) but it is impossible to draw firm conclusions in the absence of directly controlled tests to establish the influence of these factors. Christensen et al. (2021) studied discrimination of buckets which varied in size, shape and pattern by horses that were some of the youngest used in cognition research, just 10–12 months. These Danish warmblood horses lived in groups and readily learnt the task, with a 91% success rate.

McCall (1989) investigated the impact of body condition on discrimination learning performance using bucket colour as a cue. The Thoroughbred and Standardbred horses, aged from 4 to 6 years old, were divided into three groups (‘thin’, ‘moderate’ and ‘fat’) using a condition score. Horses from the ‘thin’ and ‘moderate’ groups had a higher correct response rate than horses from the ‘fat’ group, possibly because fatter horses were more easily distracted and less motivated to obtain the food reward.

In all of the studies above, the food reward was directly associated with the discriminative stimuli (i.e. food in the bucket) whereas Evans et al. (2024a) tested horses to discriminate between a black and a white card. The cards were presented in front of the horse by an experimenter and the horse had to touch one of them with its muzzle. If the answer was correct, the experimenter blew the whistle before rewarding the horse with food. Then horses were trained to a reversal learning, where the previous distractor (non-rewarded card) became the target. This difference of method and other contextual factors do not allow a direct comparison to other studies.

Unfortunately, two studies on the discrimination between two signs with a symbol (cross or round) in black on a white background (Gabor and Gerken 2010; Briefer Freymond et al. 2019) or between the same symbols presented as white on a black background (Briefer Freymond et al. 2019) used horses of various breeds which does not allow further investigation of the impact of breed. Gabor and Gerken (2010) tested four ponies of various breeds and found that none of the three ponies managed to reach the 80% correct response criterion, the fourth pony having been excluded (not having learned to walk to the apparatus and use it). However, Briefer Freymond et al. (2019) found all subjects were successful. In addition, horses that performed crib-biting stereotypies were not impaired by their stereotypy on either discrimination or reversal discrimination, unless they were prevented from crib-biting. Unlike the previous studies, in addition to varying the shape of the symbols, Tomonaga et al. (2015) varied their size. In the case of circles, the ponies were able to distinguish between two circles with a 14% difference in size, but they found it more difficult to discriminate between relatively similar shapes such as the ‘O’ and the ‘D’. Schubert et al. (2022) obtained similar results in a task where horses had to discriminate different letters of the Latin alphabet. The mean discrimination accuracy for the five letters presented against each other during the transfer test was of 80.4%. With the exception of the male, all horses were able to discriminate the letters ‘O’, ‘B’ and ‘Z’ from ‘X’ and to move to the transfer test. Females had difficulties for the following combinations: ‘O’ versus ‘B’ (78.3% correct choice) probably as the letters were curved, ‘B’ versus ‘Z’ (75% correct choice) and ‘V’ versus ‘X’ (43.3% correct choice) due to the straight line.

Some researchers also looked at the discrimination task using colours other than black. In the study of Miyashita et al. (2000), three Thoroughbreds horses had to choose between two levers depending on the colour showed in the panel centre, with blue for the left side and yellow for the right side. In the first instance, each colour/position combination was associated with a different food reward. Subsequently, the rewards were the same for the two colours or were randomly assigned. They found that the results were much better (80 to 90% of correct choice) when the combinations of colour and side were associated with a specific reinforcement, i.e. different food rewards.

Researchers also used discrimination tasks to test the ability of horses to recognise real objects and to transfer this knowledge to images of the same objects. Kappel et al. (2023) found that 27 horses out of 33 (81.82%) learned to discriminate between two dog toys. Subsequently, images of the two toys were presented on a screen (10 trials) interspersed by trials with the real toys (5 trials) but only one horse (a 14-year-old male) selected the correct image 9 out of 10 trials, performing above chance level.

The capacity of horses to discriminate quantities has been tested using artificial apples (Uller and Lewis 2009), real apples (Balestrieri et al. 2024) or geometric symbols (Gabor and Gerken 2014). The first and last experiments highlighted that when the task became more complex, i.e. large numbers of stimuli (Uller and Lewis 2009) or presence of different symbols (Gabor and Gerken 2014), the horses were less able to discriminate. Moreover, in the study of Gabor and Gerken (2014), three Shetland ponies had more difficulty discriminating heterogeneous than homogeneous symbols. Balestrieri et al. (2024) found horses failed to discriminate quantities due to a left side bias, regardless of task difficulty. Indeed, most horses chose the left side on more than 2/3 of trials, irrespective of the position of the largest quantity, and this was regardless of management background (stabled and ridden, or herd-living and non-ridden). Moreover, neither the sex nor age affected the horses’ performance.

A second group of studies has employed visual symbols as discriminatory stimuli to help horses choose between other outcomes, for example whether they want a blanket on or off depending on temperature (Mejdell et al. 2016). All the horses (100%) were able to discriminate the symbols to communicate their preference to the handler depending on the weather and the results were biologically appropriate, in that the horses indicated a preference for a blanket when the weather was bad. The horses used in this experiment were females and geldings of various breeds (10 breeds) from 3 to 16 years old.

A third group of studies has examined the discrimination abilities of horses using more ecologically relevant social stimuli, including visual (Ragonese et al. 2021), auditory (Cruz-Becerra et al. 2009), olfactory cues (Hothersall et al. 2010; Marinier et al. 1988; Péron et al. 2014) of conspecifics, as well as emotional cues from conspecifics and from humans. For the visual discrimination study, researchers investigated if female and male Franches-Montagnes horses (mean age: 11.2 years) were able to perform intra- and inter-species individual discrimination by using two-dimensional images of unfamiliar faces of horses, cows, donkeys, pigs and sheep (Ragonese et al. 2021). They found that 80% of the horses could discriminate between a horse face and the face of another species, but not between two horse faces, or two “other-species” faces.

Two, five-year old Colombian ‘fine-step’ horses were used in a study of auditory discrimination (Cruz-Becerra et al. 2009) where correct responses resulted in access to a food via an operant response. In pairwise tests, both horses were able to discriminate the sound of their unique step from the sound of other gaits such as trot or gallop.

Most studies on olfaction discrimination from samples of conspecifics have been conducted on horses living alone in stables (Hothersall et al. 2010; Marinier et al. 1988; Péron et al. 2014). In these studies, ‘natural’ differences in the behaviour of the test horses, rather than operant responses, are taken as evidence of discrimination. Researchers found that American Saddlebred stallions were not able to discriminate between anoestrus and oestrus mares from urine samples, unless the samples were presented simultaneously (Marinier et al. 1988). Subsequently, an experiment was conducted on pregnant females of various breeds to determine whether they could distinguish between the scents of different individuals (Hothersall et al. 2010). Two short presentations of a urine, faecal or body odour sample from a specific individual were followed by the simultaneous presentation of two samples, one from the same individual as before and the other one from a new individual of the opposite sex. During the simultaneous presentation, mares investigated urine samples of the new individual more than the sample of the individual they had been habituated to, suggesting that olfactory cues can be used to distinguish reproductive status. However, mares did not discriminate familiar from unfamiliar faecal and body odour samples. In a second study, mares with their foals (4 months) underwent the same procedure but using urine samples from pairs of unfamiliar castrated males. Neither mares nor their foals were able to discriminate between these samples suggesting that olfactory cues are not used to distinguish individual identity within groups of similar reproductive status (Hothersall et al. 2010). A similar experiment was conducted with body odour using mares and geldings of various breeds (Péron et al. 2014). In this experiment, contrary to the previous one, horses were able to discriminate between familiar and unfamiliar conspecific from body odour. These two studies tested horses from similar range age (5–17 years: Hothersall et al. 2010 and 7–17 years: Péron et al. 2014) and did not provide any information on the breed of their subjects.

Research has also been carried out on the emotional discrimination ability of horses using either frontal photographs of horses (Wathan et al. 2016) or videos (Trösch et al. 2020c). Three facial stimuli were used by Wathan et al. (2016): positive attention, relaxation and aggression. The horses were able to discriminate between the different pictures, as indicated by a change in heart rate when presented with the face of horses in positive attention or aggressive facial expression. In addition, horses modified their behaviour according to the emotional content of the images, showing avoidance of photographs of horses with aggressive facial expression. Trösch et al. (2020c) used videos showing positive (horse groomed by an experimenter) or negative (veterinary consultation) interactions between a human and a horse. The horses reacted according to the content of the video. When the positive interaction was shown, the horses sought contact with the human holding them and showed a positive facial expression. When the negative interaction was shown, the horses had a greater increase in heart rate than when watching the positive video. In addition, their facial expression was negative. These two studies showed that horses were able to discriminate the emotions of a conspecific spontaneously (in single trials where no reward was presented and without previous training) and to adapt their behaviour accordingly (Trösch et al. 2020c; Wathan et al. 2016). However, it is difficult to compare the two studies as no information about housing or breeds was provided by Wathan et al. (2016).

With domestication, humans have become very present in the life of horses and researchers are interested in the ability of horses to discriminate between images of human faces presented on photos (Stone 2010) or videos (Lansade et al. 2020b) and between the human emotions being presented (Sabiniewicz et al. 2020; Smith et al. 2018). Stone (2010) found that horses could discriminate between unrelated individuals, twins and identical twins and could transfer this facial recognition to field tests in the presence of the photographed people. Surprisingly, horses needed fewer days (4 to 11) to reach the criterion (success on 80% of the trials) for identical twin discrimination than for fraternal twin discrimination (21 to 22 days). In another study, Lansade et al. (2020b) investigated how Welsh ponies used elements of the face (e.g. eyes and hair colour) for facial recognition. The horses were trained to identify four unfamiliar faces, presented in colour, on a screen. Then, portraits of the same people were shown with their eyes hidden, in black and white, with their face in profile, or with a different haircut. The horses were able to discriminate the faces despite these differences (63.63% of the horses had scores above chance regardless of condition). As in the study of Stone (2010), the horses were also able to make the connection between the photographs of people and the people themselves. These two experiments showed that horses do not use a single facial feature, such as the eyes, to identify a person (Lansade et al. 2020b; Stone 2010). As Stone (2010) did not provide any contextual information in his article other than the food provided to the horses, it is not possible to draw a parallel between these two studies regarding the possible influence of the breed of horses tested and their housing. More recently, Jardat et al. (2024) tested the ability of 11 female Welsh ponies to discriminate joy and sadness against other emotions (anger, disgust, fear, surprise) with food as a controlled reward. Images of pairs of human faces in different emotions (joy or sadness versus the other emotions) were presented on a touchscreen. The success rate of the ponies was higher than chance, particularly for ponies trained to respond positively to sad faces and was also influenced by the nature of the distractor emotion.

The natural spontaneous discrimination of human emotion by horses has been studied by using human non-verbal vocalisation, i.e. laughter for the positive emotion and growling for the negative emotion (Smith et al. 2018) and looking at reactions of adult (7 to 22 years), ridden horses. When the horses were exposed to a single, unrewarded presentation of negatively valenced stimuli, they expressed natural vigilance behaviour, such as freeze posture with ears forward. Horses were therefore able to discriminate between positive and negative human vocalization in the absence of any other cues. Horses can also distinguish body odours derived from humans in fearful or happy states (Sabiniewicz et al. 2020). Body odour samples from 10 human adults were presented to 21 horses (Arabian and Thoroughbreds) living in a stable alone. When presented with the odour of fear, the horses raised their heads significantly more often and for longer than when presented with the ‘happiness’ sample. These natural responses will likely have been influenced by a long history of uncontrolled contingencies between human emotion and consequence. It has not been established whether direct reinforcement would consolidate and stabilize these natural biases into discrete, reliable performance categories.

#### Learning sets, categorisation and concept formation

##### Categorisation and generalisation

Categorization and generalization allow horses to process information efficiently, apply knowledge acquired in one context to others, and to simplify complexity. Categorization involves grouping items based on their shared characteristics, generalization involves applying knowledge from one category to similar situations or items. Most equine research has focused on perceptual categorization, particularly the use of physical (mostly visual) stimulus features e.g. geometrical figures (Dougherty and Lewis 1991; Hanggi 1999; Sappington and Goldman 1994), objects of different colours and shapes (Christensen et al. 2008) or rotated (Hanggi 2010a) versions of the objects that the horses had initially learnt to discriminate. Through geometric stimuli (solid black circles) Dougherty and Lewis (1991) have shown that Quarter horses (*n* = 4) are able to learn by discrimination and to generalize the reinforced stimulus. They also investigated the phenomenon of peak shift in a task where the horses had to learn to discriminate between two black circles of different diameter: one of 2.5 cm (rewarded by food) and another one of 1.5 cm (inducing a delay of 30 s before moving to another stimulus), before generalisation testing. Two horses reached their gradients peak at 3.0 cm and another one at 3.5 cm.

Sappington and Goldman (1994) investigated if Arabian horses could acquire a rule-based category of triangularity. Initially, all 4 individuals successfully learnt to discriminate a black triangle from another black shape (square or rectangular) for food reward. Then, they investigated triangularity by presenting the same horses with pairs of stimuli: a black triangle (rewarded) and another black shape. The pairs were drawn from three different triangles (two already learned) and three other different shapes. Only 50% of the horses succeeded in this task and, among them, only one solved the last problem, which presented three novel triangles, with no prior reward history.

This notion of categorisation of geometrical figures was also investigated by Hanggi (1999), who trained two gelding horses, of different ages and breeds, to learn to select an empty two-dimensional black geometric figure (rewarded by food), compared to the same figure but with a filled centre. Once the horses reached the criterion of 10 out of 10 correct responses during two consecutive sessions of 20 trials, 15 new pairs with the same characteristics were added. The horses demonstrated the ability to categorize and generalize the previously learned category. However, horses had more difficulties with some figures than others such as the figure X for which horses need more trials to reach the criterion.

The concept of categorisation has also been investigated with three-dimensional stimuli (Christensen et al. 2008; Hanggi 2010a). Christensen et al. (2008) found that 2-year-old Danish warmblood horses generalized between novel objects of the same colour. Habituation, evidenced by a significant decrease in heart-rate and behavioural reaction, was observed when horses were repeatedly exposed to single blue object. This habituation was generalized to other blue objects of different shapes when these were presented for the first time. However, habituation to objects of a given size was not generalized to similar-sized objects of different shapes and colours. Hanggi (2010a) trained four horses (Anglo-Arabian, Arabian, Draft X) to discriminate 3 pairs of unknown objects (with each object in the pair having the same pattern or colour). Then, the objects were presented in a spatial orientation differing from that learned during discrimination (e.g., upside down and/or rotated in depth). The horses were able to recognize objects under all rotation conditions but the degree of accuracy of recognition varied between individuals and manner of rotation. Horses did not perform as well when the bottom of the object was visible rather than the top, possibly because objects were always presented with the top visible during initial training.

Researchers have also looked for innate categories by testing the ability of horses to categorise more ecologically-relevant stimuli. Nakamura et al. (2018); Trösch et al. (2019a) investigated whether domestic horses recognize human emotions cross-modally, that is, by combining different sensory stimuli (sight and hearing). In the first study, using an expectation violation paradigm, they found that horses presented with pictures of their caretaker (happy/angry) followed by their voice (gentle/scolding) looked at the speaker faster and for significantly longer time in the incongruent condition compared to the congruent one. These data were confirmed in the second study, where two animated images (1-second videos) of a woman’s facial expressions (one of joy and the other of anger) were presented simultaneously with nonverbal human vocalizations (expressing also joy or anger) to 34 Welsh pony mares. As before, the horses tended to fixate more on the image that did not match the emotion of the vocalization. In addition, behaviour and heart rate changes occurred, with negative reactions to the anger vocalization and positive ones to the joy vocalization. These results suggest that horses can match visual and vocal cues for the same emotion and may perceive the emotional valence of human nonverbal vocalizations. Controls for some non-emotional differences between vocalizations have been implemented (e.g. duration and loudness, Nakamura et al. 2018; duration, Trösch et al. 2019a) but other controls (e.g. for pitch) should be implemented before this conclusion can be fully supported.

##### Concept formation

The ability of horses to learn the concept of similarity between stimuli presented under different conditions was investigated by Flannery (1997). Three horses were trained to touch individually presented cards with geometric stimuli with their muzzles and to give responses to two stimuli taken from a series of three. Subsequently, the stimuli were presented in other configurations (such as the card placed on different surfaces), and the horses’ task was to select cards with matching stimuli. The horses were between 73% and 83% accurate depending on the test. To examine relational concepts Gabor and Gerken (2012) investigated the ability to match-to-sample in 7 ponies. Four of the ponies progressed through the training stages and were able to match trained geometric symbols to similar samples. In the final test, the same four ponies successfully transferred this ability and were able to match completely new stimuli, demonstrating a concept of “same as” or “different from”. Similar findings were found for relative size concept “larger or smaller than” when 3 horses were tested, an Arabian horse, a Paint and a Pinto Draft cross (Hanggi 2003).

Kundey et al. (2010) showed that eight geldings exposed to structured food quantity patterns learned to follow the sequence more efficiently than those exposed to patterns without such structure, running faster for large amounts of food and slower for small amounts of food. More recently, Evans et al. (2024b) highlighted that horses were able to use a model-based strategy during an inhibitory task. After three rewarded sessions, a cost was introduced, (when horses touched a card when the light was on, a 10-second time-out occurred, with the experimenter lowering the card and looking down at the floor). From session 4 onwards, the introduction of this cost significantly reduced errors, suggesting that horses can take into account the cost-benefit outcomes of their responses.

The ability of horses to form an idea of ‘a specific individual’ horse (Proops et al. 2009) or human (Jardat et al. 2023a, b; Lampe and Andre 2012; Proops and McComb 2012) can also be considered as concept formation and can be investigated using the expectation violation paradigm (Jardat et al. 2023a, b; Lampe and Andre 2012; Proops and McComb 2012; Proops et al. 2009). In the research of Proops et al. (2009), a conspecific passed in front of a subject horse before being out of sight, then a call from this conspecific or another member of the group was played from the location where the horse disappeared. The latency to respond was quicker and the total looking time was longer when the combination was incongruent, i.e. the call did not correspond to the conspecific that they had seen pass.

Using the same paradigm, Lampe and Andre (2012) exposed a group of 12 horses to trials with incongruent auditory, visual and olfactory identity stimuli or trials with congruent stimuli. The subjects responded faster, the duration of their first look at the direction of the auditory cue was longer and the horses looked more often in this direction on the incongruent trials, showing greater interest when the identity stimuli did not match. This suggests that the equines are able to integrate the multisensory stimuli of a familiar human and form a representation of the person, and maintain recognition of this person even in the absence of some sensory information. Proops and McComb (2012) also investigated cross-modal recognition of human individuals. Using a preferential gaze paradigm, horses were presented with two people and a reproduction of their voices to determine whether they were able to match the voice to the person. When the familiar individuals were presented, the subjects were able to match the sight of the familiar person with the correct voice. More recently, this paradigm has been used to test the ability of Welsh ponies to form cross-modal representations of children and adults (Jardat et al. 2023b). Two silent videos of a child and an adult pronouncing the same sentence were presented and accompanied by the sound of a child’s or an adult’s voice speaking the sentence aloud. Ponies spent significantly more time looking at the videos that were incongruent, i.e. video of a child associated with an adult’s voice or the opposite. Jardat et al. (2023a) also used this paradigm to test the ability of Welsh ponies to form cross-modal representations of human emotions. They used a similar experimental design as Jardat et al., i.e. presentation of two videos simultaneously without sound (a joyful human face or sad one) accompanied by either a joyful or sad voice. They found that horses spent more time looking at the incongruent video than the congruent one, during initial stimulus presentation. Ponies looked also more rapidly, more frequently and for longer periods of time at videos presenting a joyful human face than videos of a sad one.

Four horses (Budjonny, Italian saddle, Quarter horse, mix breeds) have also been tested for the possession of a concept of “self”. Using a mirror self-recognition test, researchers painted a coloured cross on the horse’s cheek and compared responses to a sham condition where no mark was applied (Baragli et al. 2017a). Although the horses showed more response to the uncovered mirror than a covered mirror, suggesting they found some of the visual aspects of reflection incongruent, there was no consistent evidence that they responded differently when they were marked or unmarked.

Researchers also investigated the ability of horses to use behaviours (Lovrovich et al. 2015) or knowledge of humans (Ringhofer and Yamamoto 2017) to find a hidden source of food. The ability of horses to understand, remember and use human-provided directions in a delayed three-choice task was tested by Lovrovich et al. (2015). Twelve horses had to find a piece of carrot hidden under one of three upside-down buckets after seeing the experimenter hide the food (experimental group) or not (control group). Results showed that horses can change the decision-making strategy from reliance on human-given cues to ignoring them to favour speed (e.g. go to the bucket previously rewarded or explore more buckets). Ringhofer and Yamamoto (2017) studied whether eight horses were able to vary their behaviour based on their keepers’ knowledge of where food was hidden. The horses could not directly access hidden food but they had experience that keepers were likely to share located food. The horses’ signalling behaviour increased (visual and tactile signals) significantly under conditions where the horses had information about food location, but the keepers did not. These results suggest that horses modify their communicative behaviour towards humans based on their assessment of the humans’ knowledge state.

##### Abstract reasoning

Abstract reasoning regarding the physical properties of objects has been investigated through the use of food (Haemmerli et al. 2018). Horses of different breeds (Arabian, Freiberger and Shetland ponies) were not able to reason intuitively about the physical properties of objects, being unable to distinguish between two opaque screens, one only one of which had a suitable size and orientation to conceal a food reward. Horses were only able to make this discrimination after training.

Abstract reasoning has also been investigated with a problem-solving task. Esch et al. (2019) found that 25% of the horses were able to understand the functioning of a dispenser and to eat all the food it contained without having prior information about how to operate it.

#### Spatial cognition

The ability of horses to use information from their environment to access a resource has often been tested through detour tasks (Baragli et al. 2011, 2017b; Briefer Freymond et al. 2020; Haag et al. 1980; Osthaus et al. 2013). In these studies, a detour task was, for example, constructed as a learning maze with two exits, one on the right and one on the left (Haag et al. 1980), a barrier open at one end (Osthaus et al. 2013), a fence with an opening (Briefer Freymond et al. 2020) or a symmetrical and asymmetrical U-shaped barrier (Baragli et al. 2011, 2017b). In all studies that used a bucket of food for the reward, the horses generally succeeded in the task. However, it is not possible to determine if horses were quicker to detour in one of these studies as Briefer Freymond et al. (2020) did not give any information about the time taken to succeed by each horse, Osthaus et al. (2013) only gave the mean time to achieve the task per equid species and Haag et al. (1980) gave the mean latency to succeed for each horse (but the horses did not all have the same number of trials). Haag et al. (1980) found that, in less than 10 trials, 90% of the ponies were able to go directly to the open exit behind which the food was located. When the detour task was to go around a fence to get to the bucket containing food, Osthaus et al. (2013) found that all horses (100%) successfully completed the detour task and moreover, the average time taken by the horses to solve the detour task decreased rapidly with each trial. In these studies, there is little chance for contextual factors to play a role as the tasks seem rather ‘simple’ with all horses reaching the goal. However, adding complexity to a spatial task e.g. by adding a reversal condition, increased the variation of performance outcome. After a maximum of four repetitions, the opening in the barrier changed sides in the study of Osthaus et al. (2013). Following this change, on the first trial, 83.33% of the horses moved to the former position of the opening. The authors did not give information about the two horses which did not succeed on the first trial. By the end of the third trial, all horses (100%) were able to move correctly to the exit and the time spent on the trials decreased.

Variation of performance between individuals can be revealed not only by adding complexity to a spatial task but also by taking a greater range of performance measures. Baragli et al. (2011, 2017b) found that when horses had to make a detour of a symmetrical and asymmetrical U-shaped barrier, they were all able to perform these detour tasks, but not in the same way. Some horses always chose the same side even if the route chosen was not the shortest, while others took the shortest route (Baragli et al. 2011, 2017b). However, the researchers (Baragli et al. 2017b) found that the horses performed the task more quickly during the trials, which was not the case in the previous study (Baragli et al. 2011). The similar testing procedures raises the possibility that the results indeed reflect breed differences rather than differences in methodology. The horses used in these two experiments all lived in groups on pasture and were fed ad libitum. Moreover, they were approximately the same mean age (9.5 years for Baragli et al. 2011 and 10.8 years for Baragli et al. 2017b). However, different breeds of horses were used: Italian saddle horses (Baragli et al. 2011) and Standardbred Horses (Baragli et al. 2017b). It is possible that Standard bred horses learn faster than Italian horses or were more motivated by the presence of food. More recently, Janczarek et al. (2023) have used a real maze to test spatial learning of Hucul mares and found that all the mares (100%) successfully completed the maze.

Although most researchers have used a detour task to study equine spatial cognition, a wooden grid (ten columns and four rows) presented vertically against a wall has also been employed (Mal et al. 1993). Among 14 Arabian foals (4.5 months of age) tested, the ones which received the presentation of the reward location by an experimenter on the first day had a shorter mean distance from the reward than the others (indicating that only one presentation can enable this type of spatial learning. They also made more visits to the learning apparatus. However, foals showed separation anxiety during testing (increased locomotion and vocalisation) that may have affected the results. A recent study tested the ability of female Welsh ponies, living in groups in an indoor barn, to solve visible and invisible displacement tasks (Trösch et al. 2020b; see Table 3). For the visible displacement task, a pony was first placed in front of two empty buckets. The experimenter put food in one of them, in front of the pony, and then closed the two buckets with a lid. The pony had to touch the bucket containing the food with its nose. A similar experiment was subsequently conducted with three buckets. In these tasks, the experimenter’s last movement correlated with food location. When looking at ponies who obtained a score of at least 80% or more, only 31.25% of the ponies were successful when tested with two buckets compared to 90% when tested with two cups. They were less successful when tested with three buckets (0%) or cups (14.29%). During a final invisible displacement task, the experimenter put food in one of the buckets before closing the lid but then changed the position of the buckets in front of the pony. The pony had to find the location of the bucket containing the food. Ponies were not able to follow movement of the food (only 12.5% of the ponies tested with the buckets and 33.33% with the cups obtained a score of at least 80% or more). Researchers mentioned that in the latter task with the cups, the only pony out of three which succeeded in the control and test trials might have used an associative rule as, when the cup was moved from the middle position to an outer position, the pony did not exceed chance level.

#### Social learning

Social learning refers to learning that is facilitated or enhanced by observation or interaction with a more knowledgeable or skilled conspecific or from an individual of another species. To ascertain whether social learning occurs in horses, a typical procedure has been to expose individual ‘observers’ to the behaviour of a trained conspecific and compare their learning performance with that of naïve controls. Variations of this basic procedure have been used by researchers through different tasks such as: visual discrimination task (Clarke et al. 1996), learning an instrumental task (Ahrendt et al. 2012; Lindberg et al. 1999), diversion tasks (McVey et al. 2018; Rorvang et al. 2015b;) and crossing a new surface (Rorvang et al. 2015a).

Clarke et al. (1996) tested social learning using a task that required the selection of a food bucket based on its colour and pattern (pattern cues). The subjects were aged 2 to 20 years and of various breeds (Appaloosas, Thoroughbreds, Thoroughbreds cross, Welsh cobs and small ponies). The demonstration by a pre-trained unfamiliar horse had an effect on the subjects’ behaviour. On the first trial, the average latency to approach (18 s) and the average latency to eat (35 s) were significantly lower than those of the horses in the control group (respectively 119 and 181 s). However, this demonstration had no effect on the choice of bucket demonstrated, even after 10 trials. In subsequent work on social learning of an instrumental task, researchers used demonstrators familiar to the observers (Ahrendt et al. 2012; Lindberg et al. 1999). Both tasks consisted of opening an operant device by pressing a foot pedal (Lindberg et al. 1999) or pushing it with the muzzle in order to obtain a food reward (Ahrendt et al. 2012). These studies thus suggest socially-mediated habituation but not observational learning. In terms of breeds, both studies used horses of various breeds (Ahrendt et al. 2012; Danish Warmblood, Danish Sports Pony, Holsteiner and mixed breeds; Lindberg et al. 1999; Arab, Part Bred Arab, Appaloosa X, Cob, Haflinger, New Forest, Pony type, Welsh Cob, Welsh Sec. C, Thoroughbred, Thoroughbred X, Warmblood).

Social learning has also been investigated using spatial detour tasks. In such tasks, subjects are required to find their way around an obstacle, e.g., a barrier or a fence, to reach a target. In social learning studies, subjects’ success in reaching the target can be assessed after the correct route has been demonstrated by a human or conspecific. Rorvang et al. (2015b) used Icelandic horses housed in a group on pasture and tested them on a detour task in which horses had to find their way out of a square arena comprised of fences with one opening. The demonstrator was either the same age as the observer (3 years old) (experiment 1 and 2), or an older horse, 9 years (experiment 3). In the first two experiments, the researchers found no social learning (in both groups, 72.73% of horses succeeded the task after all three trials). However, in the second experiment, where the detour task was simplified, the observers performed better (63.64%) and faster (median ~ 25 s) than the control group (27.27%; median ~ 45 s) in their first trial. This experiment was repeated with an older demonstrator (experiment 3) who lived with them in the pasture. The horses lived with the demonstrator for a month longer in experiment 3 than in experiments 1 and 2, but this, and the age of the demonstrator, did not have a positive impact on social learning (66.67% of horses succeeded the task after all three trials regardless of the experimental group). McVey et al. (2018) also used a familiar leader demonstrator. Horses from various breeds (cob cross, Welsh cross, native cross, New Forest cross) also lived in groups in the pasture as in the study by Rorvang et al. (2015b). But unlike the latter, McVey et al. (2018) included another control group where the observer did not see the demonstrator perform the task or only saw it eat from the bucket on the other side to control for social facilitation. Like Rorvang et al. (2015b), they found no difference in task performance. It is possible that the task design was too easy. Indeed, the control group, without demonstration and other horse eating from the bucket, had a high success score (80% of horses had their 5 trials correct). In addition, there may have been learning over the course of the trials as the horses’ latency to solve the task decreased significantly between trials 1 and 2.

The experiments described above focused on ways of finding hidden food. Rorvang et al. (2015a) were interested in a task that involved overcoming fear responses. Young Icelandic horses (*n* = 22) aged 3 years and living in a group in a paddock were used to investigate the effect of a demonstrator when crossing a new surface (a white linen cloth). The researchers found no difference in task success between horses who had watched a demonstrator cross the surface or the ones that only saw the demonstrator eat from the other side of the novel surface (control group), all the horses (100%) crossed the surface on the first trial, suggesting that the task did not instil fear as the researchers had expected. However, horses that observed a demonstrator had a lower mean heart rate than control horses.

Researchers have long been interested in intra-species learning but only recently have they begun to investigate the potential role of human demonstrators. As in the previous detour task experiments, Burla et al. (2018) and Henriksson et al. (2019) were unable to demonstrate social learning, perhaps due to the use of an unfamiliar demonstrator. This inter-species learning from humans by horses has also been investigated using a task that required horses to open a feeding apparatus (Bernauer et al. 2020; Rorvang et al. 2020; Schuetz et al. 2017). Rorvang et al. (2020) did not find evidence supporting social learning by Icelandic horses (60% of the horses who received a partial or full human demonstration solved the task against 40% for those who did not). However, Schuetz et al. (2017) and Bernauer et al. (2020), who tested horses of various breeds, found the opposite result (67% and 75% success, respectively). A possible reason for this result could be the difference of breeds, as Rorvang et al. (2020) used only one breed, whereas in the other two studies there were at least three different breeds of horses. However, this possibility cannot be confirmed as information regarding housing conditions was not provided, and could be a possible influence.

#### Memory

Working memory has been investigated using a delayed-response task (Hanggi 2010b; McLean 2004; Valenchon et al. 2013a). This task consists in choosing between two food locations. An experimenter put food in one of the two buckets. After 0, 5, 10, 15, 20, or 30 s, the horses were released to choose the bucket filled with food. McLean (2004) found that after a 10-second delay, the majority of horses were unable to make the correct choice (although 3 horses out of 12 were able to solve the problem). However, Valenchon et al. (2013a) found a contradictory result as in their experiment horses were able to remember the bucket containing food after a 20-seconds delay. Hanggi (2010b) showed that one horse (an Arabian 10-year-old gelding) was successful after a 30-second delay. In these two studies, the breeds of horses tested were not the same (McLean (2004): Andalusian/Thoroughbreds crossbreds, Australian stock horses, Thoroughbreds and one Welsh pony; Valenchon et al. (2013a): Welsh ponies). Whishaw and Burke (2021) used an ecologically more relevant situation for the horses than the previous studies which were using concentrated food whereas horses are grazers, testing whether five horses of various breeds, aged 5 to 14 years old, could remember the location of a pile of dung after a short delay of 1–5 min and after a long delay 30–60 min. After both delays, the horses were less likely to reinvestigate the already smelled dung pile.

Longer-term memory has also been explored by many authors through various instrumental tasks: opening of a wooden chest (Le Scolan et al. 1997; Wolff and Hausberger 1996), walking backward on command (Valenchon et al. 2013b) and crossing an obstacle after a bell ring (Valenchon et al. 2013b). Different breeds, French saddlebred (Wolff and Hausberger 1996) or various breeds (Le Scolan et al. 1997), were able to remember the instrumental task four weeks later, (100% of success at the first trial and 94% of success within the first three trials, respectively). The Welsh ponies, living in groups, were even able to remember different tasks 22 months after acquisition (Valenchon et al. 2013b). The horses were also tested in a quantity discrimination task using a computer device (Gabor and Gerken 2018). Stimuli were presented on a screen and each subject horse had to press one of the two buttons below this screen to indicate its decision. After one year, three group-living Shetland ponies were not able to recall the discrimination task. Long-term memory has also been tested over periods ranging from 6 to 10 years between tests. Experiments were conducted on horses of various breeds (Arabian, Paint, Pinto Draft X) living outdoors (Hanggi and Ingersoll 2009). The first experiment was a discrimination task with five sets of stimuli; after 6 years, the two 13-year-old gelding horses were able to remember four of the five sets. The second one was a categorization task which was repeated 10 years later. The Paint gelding horse was able to successfully achieve this task even after this long period of time and to apply the concept of categorisation to novel stimuli. The last experiment was on one Arabian gelding horse tested on relative size concept. After 7 years, he was still able to select stimuli based on their sizes and to applied it to novel sets of stimuli. Two studies on young horses also looked at the influence of past interaction with humans on the horse’s memory retention. The horses (1-year-old for Sankey et al. 2010 and 3-year-old for Lansade et al. 2020a), lived in a group outdoors (except during bad weather conditions). The horses had to remember a human (experimenter: Sankey et al. 2010 or caretaker: Lansade et al. 2020a) they had interacted with 6 months before. Sankey et al. (2010) found that regardless of the familiarity with the experimenter, horses trained with positive reinforcement approached the experimenter and accepted being touched on the shoulder more quickly than horses from the control group which were trained without positive reinforcement; they also spent more time at a close distance to the experimenter. The horses were thus able, 6 months later, to remember the positive interactions exchanged with the familiar experimenter during training and generalise this positive relationship to an unfamiliar experimenter. Lansade et al. (2020a) found that horses could even recognise a photo of a familiar person they had last interacted with 6 months previously. In both studies, although the horses were not of the same breed (Anglo-Arabian and French Saddlebred for Sankey et al. (2010), and Welsh ponies for Lansade et al. 2020a), they were housed in similar conditions.

A recent study looked at the impact of housing conditions (lighting levels and bedding height) on sleep duration and its impact on spatial memory (Greening et al. 2021). Researchers did not find an effect of degraded housing but, they found that the time taken to complete the task was greater. The authors point out that the result might have been different if the task had been more cognitively challenging.

## Discussion

This review provides an overview of the broad range of cognitive abilities that have been investigated in horses, particularly in the areas of discrimination learning, learning sets, categorisation and concept formation, spatial cognition, social learning, and memory. Among these, studies on discrimination and categorization/learning are particularly well represented. On the one hand, this is in line with similar reviews focusing on other animal species, such as dogs (Bensky et al. 2013), cats (Vitale Shreve and Udell 2015), pigs (Gieling et al. 2011), and goats (Mason et al. 2024; Zobel and Nawroth 2020), which also report a wealth of discrimination studies. On the other hand, some areas appear to be understudied in horses compared with other domesticated species. These include object permanence (dogs: Gagnon and Doré 1994; Miller et al. 2009; Pasnak et al. 1988; cats and dogs: Triana and Pasnak 1981; goats: Nawroth et al. 2015; cats: Doré 1986; Dumas 1992; Goulet et al. 1994; but see horses: Rørvang et al. 2021), physical cognition (e.g., means-end understanding: dogs: Osthaus et al. 2005; Range et al. 2012; cats: Whitt et al. 2009), social learning from conspecifics (pigs: Nicol and Pope 1994; Oostindjer et al. 2011; Veit et al. 2017, 2023; dogs: Pongrácz et al. 2004; but see horses: Rørvang et al. 2015b), maze tasks (holeboard task in pigs: Arts et al. 2009; Bolhuis et al. 2013; eight-armed radial maze in dogs: Craig et al. 2012; Macpherson and Roberts 2010; water maze in pigs: Hammell et al. 1975; sand maze in dogs: Salvin et al. 2011; spatial middle identification in chicks: Tommasi et al. 1997), contra-freeloading (chickens: Lindqvist et al. 2002; goats: Rosenberger et al. 2020), and perspective taking (dogs: Bräuer et al. 2004; ravens: Bugnyar et al. 2016; Schloegl et al. 2007). Closing these research gaps through future studies would not only advance our knowledge on horse cognition but would also allow researchers to more thoroughly investigate the evolutionary origins of these cognitive abilities via cross-species comparisons.

Interestingly, among the studies reviewed, some report effects of individual characteristics, such as age, sex, breed, and management conditions on horse cognition (e.g., Mader and Price 1980; Mejdell et al. 2016; d’Ingeo et al. 2019; Lindberg et al. 1999). Although results are highly variable across studies and cognitive domains, some of the effects are in agreement with evidence from other domesticated species. Concerning breed differences, for example, Wobber et al. (2009) found that working task dog breeds performed better at following human pointing than those selected for companionship. Similarly, Dobos and Pongrácz (2023) showed that cooperative working dog breeds learn more efficiently from human demonstrations in a detour task than independent working dog breeds, contradicting an earlier study, in which no breed effects in inter-specific social learning were detected (Pongrácz et al. 2005). In another study, not just an effect of breed but also an effect of dogs’ size on their ability to use human-given cues was found (Helton and Helton 2010).

Age effects have also been reported for other species. These are, however, often ambiguous for two important reasons. First, even for cognitive domains for which increased performance during development can easily be predicted, results are contradictory. For example, animals’ ability to follow human cues should increase across development due to prolonged exposure to humans. Nevertheless, while Duranton et al. (2017) found older dogs to be more responsive in an A-not-B task, in which a human demonstrator hid food behind one of two screens, Pongrácz et al. (2019) report that *younger* cats more successfully read human cues than did older cats. Many other studies failed to find any effect of age at all (e.g., Kaminski et al. 2012; Pongrácz et al. 2005). Second, age effects, particularly effects of maturation, are difficult to interpret. On the one hand, domestication has led to alterations of development, particularly neoteny, i.e., an extended period of immaturity in domesticated compared with wild animals (Price 1984; Wilkins et al. 2014). Potentially, such differences might also occur between breeds of the same species differing in selection histories. On the other hand, animals’ cognitive development, apart from potentially occurring at different timepoints among breeds, is difficult to measure. Some studies have tried to measure cognitive development within species. In dogs, for example, studies employing a cognitive test battery found that puppies performed worse than adults (Bray et al. 2020) but improved at around 21 months of age (Bray et al. 2021). Nevertheless, it is still difficult to pinpoint stages of cognitive development, such as cognitive maturity, and to compare timepoints among (human and non-human) species or among precocial species, such as the horse, and altricial species such as dogs and cats. For instance, precocial species’ cognitive development might rely more heavily on cognitive priors, e.g., innate predispositions, whereas altricial species need to acquire most of their skills through trial-and-error learning (Vallortigara 2024; Versace et al. 2018). Finally, within and between species, processes other than maturation might be at work, such as aging.

Despite existing within-study effects of subject characteristics in horse cognition studies, drawing conclusions from between-study comparisons for this review was very complex. This is surprising, given that the variety of breeds and ages represented in the studies reviewed was greater than for some other species (e.g., for a review on pigs see Gieling et al. 2011). However, not all studies reported the subjects’ characteristics sufficiently. In addition, methodologies were often not sufficiently comparable across studies. For instance, the various detour studies conducted with horses differ in set-up (e.g., a straight barrier (Osthaus et al. 2013) or a U-shaped fence (Baragli et al. 2011)) and in the variables recorded (e.g., latency (Haag et al. 1980) or only success (Briefer Freymond et al. 2020)). Given that the set-up can have a decisive impact on the difficulty of the detour task and animals’ success (e.g., with outward detours being easier than inward detours (Kabadayi et al. 2018)), a stricter alignment of methodologies is called for to be able to draw conclusions about the influence of age, sex and other characteristics from between-study comparisons.

Gauging the effect of management conditions on horse cognition, within and between studies, is particularly difficult for two important reasons. On the one hand, not only current management conditions, those measurable in the studies, but also past management conditions, especially during cognitive development, might be decisive to cognitive performance (but see Luo et al. 2019). In other species, ample evidence points to the important role of experience during sensitive periods (Bornstein 1989) in the development of behaviour and cognitive abilities (e.g., rats: Alves et al. 2022; dogs: Freedman et al. 1961; goats: Toinon et al. 2021). On the other hand, the average individual horse may change its owner and/or location more frequently than other domesticated animals such as dogs or cats. This makes it even more difficult to reconstruct and measure a horse’s past management experience and to understand the conditions they were exposed to during development. All these factors might explain why only one of the studies considered here found an effect of management practices on horse cognition.

In addition to management conditions, such as turnout, feeding regime or exercise, also horses’ experience more broadly can determine their success in cognitive tasks. That is, knowledge gained in past situations could help horses solve specific tasks. For example, Albiach-Serrano et al. (2012) found that pigs’ success in a physical cognition task seemed to depend on their experience with physical environments, and that their success in a pointing task depended on their experience with humans. Horses vary greatly in their individual experience, with humans, the physical environment and other horses, but these differences are often difficult to assess in the subjects of cognitive studies. Hence, whenever possible, subjects’ experiences should be reported as completely as possible to better place their cognitive performance into context.

Finally, the distribution of breeds, ages, and sexes in horse cognition studies may not sufficiently match that of the general horse population. That is, some breeds may be over- or under-represented and often cognition studies include no or only few stallions in their sample. Reliable data about horses’ characteristics are only available for a limited number of countries, such as Great Britain, where stallions make up 1.6% of the registered horse population (Wylie et al. 2013). Despite their limited representation among leisure, sport, and work horses (Pietrzak et al. 2013; Sobotková et al. 2022; Spence et al. 2018a, b), the inclusion of intact males is essential for discovering sex differences in horse cognition (e.g., hormonal levels might be more indicative than the binary variable sex Maney 2016; cf. rodents: Koszałka et al. 2023). In some areas of horse cognition research, the issue of representativeness is more systematic. That is, research on some aspects of horse cognition has mainly been advanced by single research groups with their respective areas of expertise and study populations (for a critical review of biases in research populations see Webster and Rutz 2020). For instance, many of the intra- and inter-specific social learning studies with horses have been conducted by one research group, using only Icelandic horses (Rørvang et al. 2015a, b, 2020). Analogously, another group, working exclusively with similarly-aged Welsh pony mares, contributed most of what we know today about horses’ social cognition employed in interactions with humans (Gouyet et al. 2023; Jardat et al. 2022, 2023a, b, 2024; Lansade et al. 2020, 2021). The field of equine cognition will benefit greatly if the generality of these important findings is tested by other groups working with different breeds of horse.

One way of achieving this (i.e., sufficient standardization of methodologies and increased variety/representativeness of the study populations), would be a *Many Horses* project. Looking at successful collaborations focusing on other species, such as *Many Dogs* (Alberghina et al. 2023), *Many Birds* (Lambert et al. 2022) or *Many Babies* (ManyBabies Consortium 2020; Schuwerk et al. 2021), potential advantages of a Many Horses project can be identified. That is, a larger sample size can be obtained and previous studies with small sample sizes can be replicated (cf. conclusions Bensky et al. 2013). Hence, multi-lab studies could improve external validity and reproducibility of the data (Bodden et al. 2019; Würbel and Garner 2007; but see: von Kortzfleisch et al. 2022) by allowing researchers to observe whether effects persists even with high heterogeneity in the sample/subject group. At the same time, the diversity present in the general horse population could more accurately be depicted in multi-lab studies (cf. dogs: Alberghina et al. 2023), which currently is a weak spot in horse cognition research. Moreover, multi-lab studies would allow for more standardized test procedures.

Furthermore, there are some common caveats among the horse cognition studies we have reviewed. First, almost all studies using positive reinforcement training use food as a reinforcer. Given that horses are grazers, offering them concentrated treats as rewards might not be the most ecologically valid approach to testing their cognitive abilities. For other species, test procedures and reward types have already been tuned more finely to the subjects’ ecology. For instance, many cognitive studies on young chicks use imprinted objects instead of a food reward (e.g., Lemaire et al. 2021; Rugani et al. 2010; Vallortigara et al. 1998). Further, across studies, 89–94% of horses had outdoor access, while 40% of horses were at least partially kept in social isolation which itself could influence test performance. For example, Søndergaard and Ladewig (2004) found that young horses (from weaning up to approximatively 2-year-old) reared alone were slower to learn basic tasks during training and were more aggressive towards the experimenter, whereas group-housed horses were more agitated. The latter might simply be the result of the temporary social separation occurring during test for group-housed horses. Therefore, horses’ true cognitive abilities might be masked either by the effects of extended social isolation, in the case of singly-housed horses, or by the stressfulness of temporary separation, in the case of group-housed horses not gradually familiarized with the experimental procedure.

In conclusion, three main recommendations for future horse cognition studies emerge from the present review. First, we consider it essential that researchers better report subject characteristics in scientific publications, to allow for an investigation of the factors shaping horses’ cognitive abilities. Second, we advocate more standardized methods and procedures across studies. Specifically, multi-lab studies using standardized procedures, i.e., Many Horses projects, could facilitate the systematic assessment of cognitive abilities across contexts/labs. Finally, we want to encourage the research community to broaden the range of cognitive abilities studied in horses. The present review strongly suggests that all of these steps will be necessary to gain a more complete overview of horses’ cognitive abilities, of the individual and contextual factors that shape them, and to elucidate how they compare to other species.

## Data Availability

No datasets were generated or analysed during the current study.
